# Adaptation to pH stress by *Vibrio fischeri* can affect its symbiosis with the Hawaiian bobtail squid (*Euprymna scolopes*)

**DOI:** 10.1099/mic.0.000884

**Published:** 2020-01-22

**Authors:** Meagan Leah Cohen, Ekaterina Vadimovna Mashanova, Sveta Vivian Jagannathan, William Soto

**Affiliations:** ^1^​ College of William & Mary, Department of Biology, Integrated Science Center Rm 3035, 540 Landrum Dr., Williamsburg, VA 23185, USA

**Keywords:** Microbial experimental evolution, host-microbe interactions, symbiosis, mutualism, bioluminescence

## Abstract

Many microorganisms engaged in host-microbe interactions pendulate between a free-living phase and a host-affiliated stage. How adaptation to stress during the free-living phase affects host-microbe associations is unclear and understudied. To explore this topic, the symbiosis between Hawaiian bobtail squid (*Euprymna scolopes*) and the luminous bacterium *Vibrio fischeri* was leveraged for a microbial experimental evolution study. *V. fischeri* experienced adaptation to extreme pH while apart from the squid host. *V. fischeri* was serially passaged for 2000 generations to the lower and upper pH growth limits for this microorganism, which were pH 6.0 and 10.0, respectively. *V. fischeri* was also serially passaged for 2000 generations to vacillating pH 6.0 and 10.0. Evolution to pH stress both facilitated and impaired symbiosis. Microbial evolution to acid stress promoted squid colonization and increased bioluminescence for *V. fischeri *, while symbiont adaptation to alkaline stress diminished these two traits. Oscillatory selection to acid and alkaline stress also improved symbiosis for *V. fischeri *, but the facilitating effects were less than that provided by microbial adaptation to acid stress. In summary, microbial adaptation to harsh environments amid the free-living phase may impact the evolution of host-microbe interactions in ways that were not formerly considered.

## Introduction

### The effects of microbial stress evolution on host-microbe interactions

In nature, microbes can confront arduous environmental conditions that are stressful [1, 2]. Examples include heavy metal toxicity, extreme temperatures, and ultraviolet light [3]. Harsh pH environments can also impede microbial physiology. Hence, microorganisms need to evolve the capacity to tolerate challenging conditions and constantly changing environments [4]. For microbes pendulating between a free-living stage and a host-associated phase, an intriguing research question is how stress evolution by free-living cells will impact the capability to colonize the host [1]. This query is pertinent to the entire spectrum of host-microbe associations: pathogeneses (parasitisms), commensalisms, and mutualisms [5]. Cross-connection between microbial stress responses and host colonization determinants has been reported previously in host-pathogen interactions [6–8].

### The sepiolid squid-* Vibrio fischeri* mutualism

Sepiolid squid (*Euprymna* spp.) and the marine bioluminescent bacterium *Vibrio fischeri* form a symbiosis that has become a model for investigating associations between animal hosts and bacteria, since both partners can be maintained independently of each other in the laboratory [9]. *V. fischeri* can be grown in monoculture, while the cephalopod host can be raised axenically [10]. The Hawaiian bobtail squid (*Euprymna scolopes*) was used for this particular study [11]. Within the squid host, *V. fischeri* cells inhabit a complex anatomical structure termed the light organ, where the bacteria are supported and sustained in a microenvironment that is abundant in nutrients relative to ambient seawater [9]. The light emitted from the bacterial symbionts is used by the squid for counterillumination. Counterillumination enables the squid to covertly maneuver, prowl, and lurk at night amid diffuse but bright nocturnal luminance, which originates from heavenly bodies (moon and stars), airglow, auroras, zodiacal light, and gegenschein [12]. Counterillumination is active camouflage utilized by organisms, where light is produced to match their backgrounds in both brightness and wavelength [13]. Counterillumination reduces the contrast of an organism’s silhouette against the surrounding environment, which enables crypsis to avoid detection by other animals. Analogous to countershading, counterillumination provides the squid with concealment, as the animal’s contour and profile are disguised [14]. Squid hatchlings emerging from their eggs possess axenic light organs, which are colonized within hours by free-living *V. fischeri* from ambient seawater [9].

Daily at dawn, 90–95 % of the symbionts are expelled or vented from the light organ to the ocean milieu by the squid [15]. The residual bacteria that continue to occupy the light organ after venting divide via binary fission with a rapid doubling time (20–30 min), which replenishes the light organ to a full complement of symbionts by nightfall [16]. At daybreak, the squid bury in the sand and sleep the remainder of the day. The squid awaken at dusk and arise from the sand to commence their nocturnal activities (foraging for food, pursuing mates for reproduction, establishing territories or domiciles, etc.) [11]. Bacteria discharged via venting can subsist and propagate in the ocean, yet with a considerably slower growth rate (from hours to weeks) than in the light organ [16]. Vented bacteria are able to colonize succeeding generations of squid hatchlings. Interestingly, *V. fischeri* is a pervasive microbe and is found in all the world’s oceans, even occurring in marine environments where the symbiont’s animal hosts are absent [16]. Additionally, *V. fischeri* strains exist in the ocean that do not form bioluminescent mutualisms with sepiolid squid. In fact, some *V. fischeri* strains are missing the complete *lux* operon [17]. How quorum sensing operates (if at all) in such strains is unclear. *V. fischeri* can also adopt a commensal lifestyle with animal hosts, by being part of the gut microbiota for example [18].

### *Vibrio fischeri* adaptation to pH stress & extreme acid-alkaline fluctuations

Vibrionaceae mostly inhabit brackish and marine habitats in tropical, temperate, and polar climates [18]. There are a few species (e.g. *Vibrio cholerae* and *Vibrio mimicus *) that can thrive in freshwater environments. A few species are also moderately halophilic that reside in brine ponds, soda lakes, and other hypersaline aquatic habitats (Dead Sea and Great Salt Lake) [19, 20]. Vibrionaceae have been isolated from salt lakes, estuaries, coastal, surface, and pelagic waters in the ocean [21]. They have also been obtained from salt ponds, salt marshes, tidal flats, lagoons, embayments, and the deep sea. Subterranean water systems (salt aquifers) may also be inhabited by Vibrionaceae [1]. Essentially, most Vibrionaceae can inhabit any water habitat, where the salinity is at least brackish. Vibrionaceae can also sustain themselves as biofilms attached to aquatic sediments, suspended particulate matter, and the external and internal surfaces of other organisms [22, 23]. Species tolerant of freshwater can be found in ponds, streams, rivers, and lakes.

Supporting Information (Supplemental Background, available in the online version of this article) details extensively the stressful pH environments the Vibrionaceae can encounter within aquatic habitats in modern times. Vibrionaceae abound in all the aquatic environments mentioned here, where the pH conditions are described for different present-day locations. Hence, Vibrionaceae experience the accompanying pH fluctuations in these various environments. In the future, marine microbes will contend with ocean acidification due to human activities, such as the burning of fossil fuels [24]. Carbon dioxide levels have been steadily rising on Earth in recent decades, which is decreasing the pH in the oceans due to carbonic acid formation [25]. Even small pH changes can have drastic impacts on microbial communities, including the Vibrionaceae [26]. Nevertheless, how pH stress evolution in the free-living environment affects the ability of microorganisms to interact with animal hosts is poorly understood. Microbial experimental evolution is an ideal approach to address this research question, especially with microorganisms renown for engaging in host-microbe interactions. This would include the Vibrionaceae, which can be pathogens, commensals, and mutualists with eukaryotic hosts [21, 27]. The Vibrionaceae have previously been used in microbial selection studies [9, 28]. Unfortunately, there are few studies where microbial selection experiments were used to investigate the effects of pH stress evolution on host-microbe interactions [29, 30]. For the current paper, the hypothesis was tested that microbial evolution to low and high pH stress both impact host colonization. To test this hypothesis, the sepiolid squid-* Vibrio* symbiosis was utilized.

## Methods

### Strains, neutral markers, microbiological media, & buffers

*V. fischeri* EM17 (ATCC 700602) was the strain used for this study [16]. For microbial selection experiments, one needs to distinguish the evolving bacteria from the ancestral strain [31]. This is done with the use of ‘neutral markers’. Neutral markers are traits that permit the ancestral and derived (descendent) bacteria to be delineated from one another, but otherwise these traits have no effect on relative fitness for any assays conducted in the study [9]. *V. fischeri* EM17 is sensitive to the antibiotic chloramphenicol (CAMS). Chloramphenicol resistance (CAMR) was used as a neutral marker in this investigation (Table 1) [9, 32]. An isogen of *V. fischeri* EM17 that is resistant to chloramphenicol was constructed using the mini-Tn7 transposon and named *V. fischeri* EM17Tn7. The routine insertion of the chloramphenicol resistance gene as a neutral marker, into the *V. fischeri* chromosome, using the mini-Tn7 transposon has already been described in great detail elsewhere [32, 33]. In this study, chloramphenicol was used at a concentration of 20 µg ml^−1^.

**Table 1.** The ancestors and derived lines from the current study are listed *
V. fischeri* EM17 and EM17Tn7 are isogenic strains that are sensitive (CAMS) and resistant (CAMR) to the antibiotic chloramphenicol, respectively. Each was evolved for 2000 generations to the same pH selection regimes, FLM pH 6.0, 7.4, 8.0, 10.0, and 6.0/10.0 (*n*=20 each). Hence, two ancestral varieties (EM and TN) underwent 2000 generations of evolution in each pH selection regime, which led to two varieties of derived lines (EM2000 and TN2000).

FLM pH Lineage	ET (CAMS) and TN (CAMR) Varieties	Nickname
Ancestral lines	EM and TN -unevolved line	Ancestors
FLM pH 6.0 derived lines	EM2000 pH 6.0 and TN2000 pH 6.0 evolved line lower pH limit	Acid specialists
FLM pH 7.4 derived lines	EM2000 pH 7.4 and TN2000 pH 7.4 evolved line optimal pH control	Optimists
FLM pH 8.0 derived lines	EM2000 pH 8.0 and TN2000 pH 8.0 evolved line middle pH control	Centrists
FLM pH 10.0 derived lines	EM2000 pH 10.0 and TN2000 pH 10.0 evolved line upper pH limit	Alkaline specialists
FLM pH 6.0/10.0 derived lines	EM2000 pH 6.0/10.0 and TN2000 pH 6.0/10.0 evolved line fluctuating lower upper	pH generalists

There were seven different pH buffers used in this study to make microbiological media or artificial seawater (Table S1). These buffers are used for different pH ranges [34]. The first type of medium used was fortified Luria-Bertani salt (FLS: 1.0 % w/v tryptone, 0.5 % w/v yeast extract, 0.3 % v/v glycerol, 3.0 % w/v NaCl, 0.03 % w/v KCl, 0.01 % w/v disodium glycerol 2-phosphate pentahydrate, 2.0×10^−3^ % w/v FeSO_4_, and 0.1 % v/v modified 1000X micronutrient solution, 50 mM Tris, pH 7.0–9.0). Modified 1000X micronutrient solution is comprised of 3.7×10^−4^ % w/v ammonium molybdate tetrahydrate, 4.0×10^−4^ % w/v CoCl_2_, 5.0×10^−3^ % w/v MgSO_4_, 5.0×10^−3^ % w/v CaCl_2_, 2.0×10^−3^ % w/v boric acid, 2.0×10^−3^ % w/v MnCl_2_•4H_2_O, and 3.0×10^−4^ % w/v ZnSO_4_•7H_2_O [35].

The second medium utilized was fortified Luria-Bertani salt buffer mixture (FLM). FLM has the same formulation as FLS, but Tris is substituted with a buffer cocktail of aminosulfonic acids (final concentration: 10 mM MES, 10 mM HEPES, 10 mM EPPS, 10 mM CHES, and 10 mM CAPS, pH 5.4–11.0). Fortified Luria-Bertani salt with modified Sorensen phosphate (FLP) is a third medium used (supplementary Table S1). FLP has the same recipe as FLS, but Tris is replaced with an inorganic phosphate buffer (final concentration: 0.7 % w/v Na_2_HPO_4_ and 0.3 % w/v NaH_2_PO_4_, for pH 8.0; Stoll and Blanchard 1990). Increasing the temperature from 25 to 28 °C for *V. fischeri* incubation had negligible effect on the useful pH range of all the buffers in this study. Supplementary Table S2 shows the p*K*_a_ values of the buffers used in this study are not sensitive to modest temperature changes such as Δ±3 °C units [36].

Throughout this study, the same size test tubes were used (25×150 mm borosilicate glass), and 2.0 % w/v Petri agar plates were used when working with solid media. ‘Standard’ FLS, FLM, and FLP are set to pH 7.5 [16]. *V. fischeri* EM17, EM17Tn7, and their derived lines (evolved bacteria) were maintained in a −80 °C freezer in 2 ml cryovial tubes with cryoprotectant solution (final concentration: 50 % v/v liquid culture, 25 % v/v glycerol, and 3.0 % w/v NaCl). As necessary, the ancestral and derived lines were retrieved from −80 °C freezer (‘frozen fossil’ record) and streaked for isolation onto agar plates. These plate cultures were incubated for 24–48 h at 28 °C, which is the optimal growth temperature for *V. fischeri* [16]. See Supporting Information for sterile materials and aseptic techniques used in this study. Regarding all statistical analyses, standard conventions were followed for *p*-values, namely *ns*=*P*>0.05 (not significant), *=0.01 <*P*≤0.05, **=0.001 <*P*≤0.01, and ***=*P*≤0.001 [37].

### Microbial experimental evolution of *V. fischeri* to pH Stress for 2000 generations to generate acid specialists, alkaline specialists, and pH generalists

Refer to Supporting Information to see how lower and upper pH limits of growth were determined for *V. fischeri *. To generate starter cultures, single colonies (from FLM pH 7.5 agar plates) of *V. fischeri* EM17 and EM17Tn7 were separately inoculated into test tubes containing 10.0 ml FLM pH 7.5. Broth cultures were incubated for 12 h at 28 °C and 200 r.p.m. To generate serial transfers, the *V. fischeri* starter cultures (100.0 µl) were used to inoculate test tubes containing 9.9 ml FLM pH 7.5. Broth cultures were incubated for 3 h at 28 °C and 200 r.p.m. to generate log phase serial transfers. Serial transfers (100.0 µl) for each strain were used to inoculate replicate test tubes (*n*=20) containing either 9.9 ml FLM pH 6.0 (lower growth limit), pH 7.4 (optimal growth control), pH 8.0 (middle control), or pH 10.0 (upper growth limit). These were the different pH selection regimes (Table 1). The different derived lines that resulted from each of these pH selection regimes were called ‘acid specialists’, ‘optimists’, ‘centrists’, and ‘alkaline specialists’ (Table 1), respectively. The starting cell density for each strain was 5.0×10^5^ c.f.u.s ml^–1^. These test tube cultures were incubated for 12 h at 28 °C and 200 r.p.m. See Supporting Information for explanation on 12 h incubation time.

**Fig. 1.**
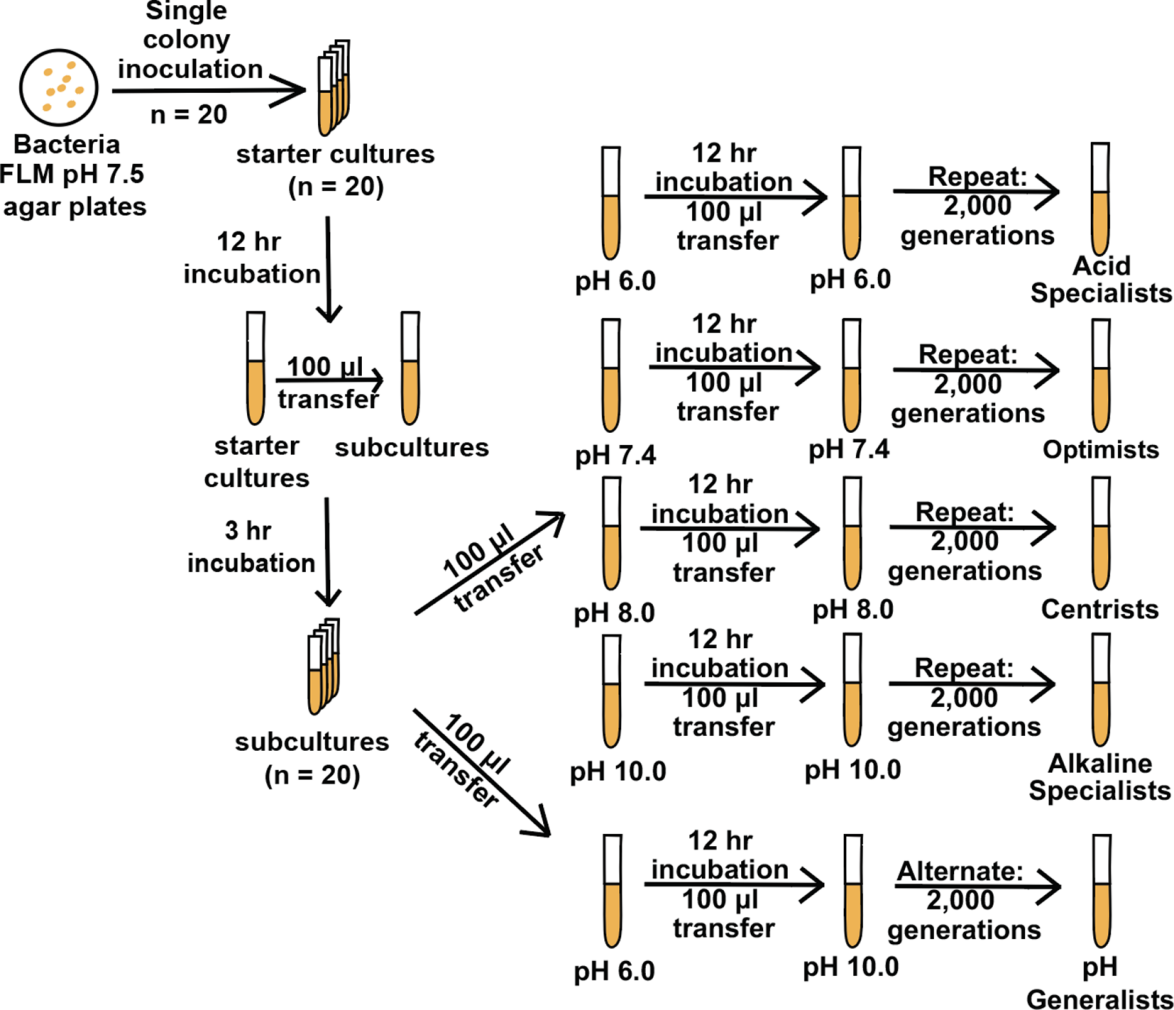
This schematic shows the different selection regimes that generated the acid specialists, optimists, centrists, alkaline specialists, and pH generalists for *V. fischeri* after 2000 generations of evolution.

After 12 h, each replicate liquid culture was serially transferred (100.0 µl) into fresh medium (9.9 ml) of its own type in test tubes (*n*=20). Thus, *V. fischeri* EM17 in FLM pH 6.0, pH 7.4, pH 8.0, and pH 10.0 were serially transferred into fresh FLM pH 6.0, pH 7.4, pH 8.0, and pH 10.0, respectively. The same was done for *V. fischeri* EM17Tn7. These liquid cultures were once again allowed to incubate for 12 h at 28 °C and 200 r.p.m. This procedure continued with 100-fold dilutions every 12 h until *V. fischeri* EM17 and EM17Tn7 both underwent 2000 generations of evolution in each of FLM pH 6.0, pH 7.4, pH 8.0, and pH 10.0 (Fig. 1). The number of generations was determined through growth curve kinetics [3, 38]. Another microbial selection experiment was conducted where both ancestral varieties experienced oscillatory selection between FLM pH 6.0 and pH 10.0 (fluctuating lower upper). This selection experiment was conducted analogously to the constant pH regimes except that the serial transfers were rotated through FLM pH 6.0 and pH 10.0 (Fig. 1). Thus, *V. fischeri* EM17 and EM17Tn7 both underwent 2000 generations of microbial evolution to temporally fluctuating FLM pH 6.0 and pH 10.0. This pH selection regime was signified by ‘pH 6.0/10.0’. The derived lines resulting from this pH selection regime were called ‘pH generalists’ (Table 1). For each of these pH selection regimes, 1.0 ml of liquid culture from each transfer was mixed with 1.0 ml of cryoprotectant solution in 2 ml cryovial tubes. Cryovial tubes were vortexed and placed in the −80 °C freezer [9]. Refer to Supporting Information on how the acid specialists, optimists, centrists, alkaline specialists, and pH generalists were grown along a pH gradient in FLM.

### Relative fitness assays

**Fig. 2.**
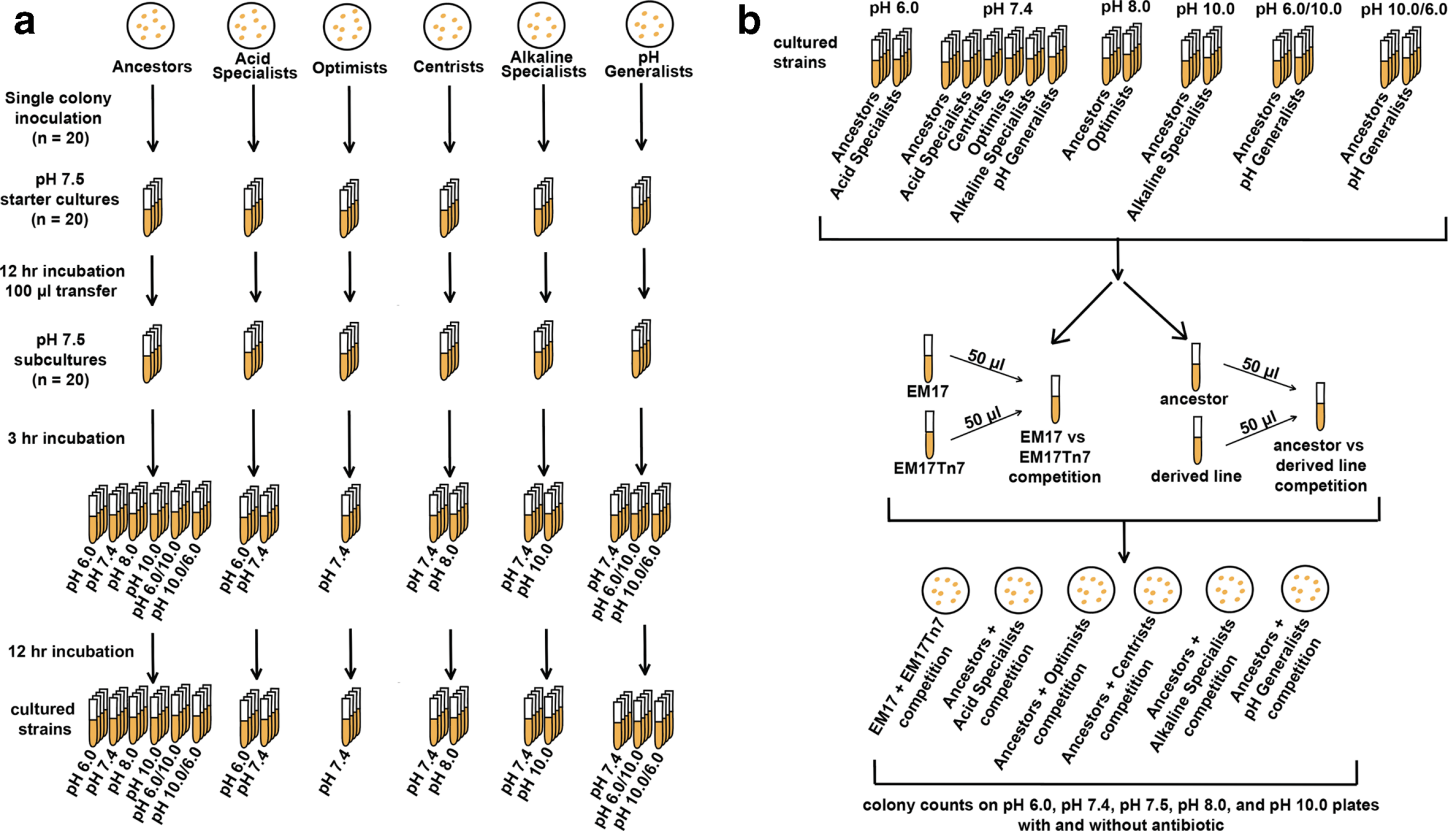
2a. This illustration outlines how the *V. fischeri* ancestors and derived lines were grown and at what pH values acclimation took place. The pH generalists involved two different acclimation schemes (pH 6.0/10.0=pH 6.0→10.0→6.0 and pH 10.0/6.0=pH 10.0→6.0→10.0). 2b. After acclimation, the ancestors and derived lines were competed against one another at select pH values. The two ancestral varieties were also competed against one another as controls, including with the pH 6.0→10.0→6.0 and pH 10.0→6.0→10.0 schemes.

The protocol described here was inspired from previous work [29, 39, 40]. Competition experiments were set up between the derived lines (bacteria evolved in FLM pH 6.0, 7.4, 8.0, 10.0, and 6.0/10.0 for 2000 generations) and the ancestors (unevolved *V. fischeri* EM17 and EM17Tn7) (Fig. 2). Competitions were arranged between a derived line and an ancestor that were oppositely marked for sensitivity and resistance to chloramphenicol. For instance, *V. fischeri* EM17 (CAMS) evolved in FLM pH 6.0 for 2000 generations (i.e. EM2000 variety of the acid specialists) was competed against unevolved *V. fischeri* EM17Tn7 (CAMR) in FLM pH 6.0, which is the lower growth limit. Similarly for the cross experiment, *V. fischeri* EM17Tn7 (CAMR) evolved in FLM pH 6.0 for 2000 generations (i.e. TN2000 variety of the acid specialists) was competed against unevolved *V. fischeri* EM17 (CAMS) in FLM pH 6.0. Similar competition experiments were conducted in FLM pH 7.4 (optimal growth control), 8.0 (middle control), 10.0 (upper growth limit), and 6.0/10.0 (fluctuating lower upper) (Fig. 2). The aim of these experiments was to examine relative fitness between the derived lines and their ancestors in the pH environments, where evolution took place for the derived line participating in the competition. In this study, the derived lines did not directly compete against one another. To control for general lab adaptation, all the derived lines were also competed against the ancestors in FLM pH 7.4.

The derived lines and the ancestors acclimatized to the environments in which they were going to compete with one another (Fig. 2a) [40, 41]. For the derived lines, cells were always inoculated into the same FLM pH medium that they evolved in. For the evolved bacteria from each of the constant FLM pH regimes (pH 6.0, 7.4, 8.0, and 10.0), each of the twenty derived lines went into a single test tube against its oppositely marked (CAMS/CAMR) ancestor (Fig. 2b). To initiate competitions between derived lines and ancestors, 50 µl of each were inoculated into test tubes containing 9.9 ml FLM pH medium at the pH in which evolution proceeded for the derived line. Thus, a 1 : 1 volumetric ratio of the derived and ancestral lines was co-inoculated into the same replicate test tube, combining for 100.0 µl total (Fig. 2b). Analogous competitions were also implemented between unevolved *V. fischeri* EM17 and *V. fischeri* EM17Tn7 in FLM pH 6.0, 7.4, 8.0, and 10.0 (*n*=20), which served as controls. To census the initial cell densities of the competitors, the test tubes were immediately mixed by vortexing, and a 50 µl sample was then taken from each test tube to be serially diluted and spread onto FLM pH 7.5 agar plates with and without chloramphenicol. As a precaution, serial dilutions were also spread onto FLM at the pH (pH 6.0, 7.4, 8.0, or 10.0) from which the 50 µl sample was taken. The test tube cultures (from which the 50 µl sample was taken) were incubated for 12 h at 28 °C and 200 r.p.m. Thereafter, the final cell densities of the competitors were censused through the enumeration of 50 ul samples onto agar plates (with and without chloramphenicol) as explained earlier. Plates for initial and final censuses of competitors were incubated at 28 °C for 24–48 h (Fig. 2b).

The initial and final cell densities were used to record the number of cell divisions that the ancestors and derived lines underwent [40]. Relative fitness differences (Malthusian parameters) were then calculated, as outlined previously [31, 41], between the ancestral and derived lines for each FLM pH medium [39, 40]. Relative fitness ratios were statistically analysed as two-tailed one-sample *t*-tests (Type 1 α error=0.05) and compared to the *H*_0_=1.00 [39, 40]. The fitness assay for the lines that were evolved in temporally fluctuating FLM pH 6.0 and 10.0 (FLM pH 6.0/10.0) was performed as outlined above except two different acclimation schemes were used (Fig. 2a), one in pH 6.0 before competition in pH 10.0 (pH 6.0→10.0→6.0, *n*=20) and another in pH 10.0 before competition in pH 6.0 (pH 10.0→6.0→10.0, *n*=20). These results were statistically analysed as described above for the derived lines evolved in constant pH. The relative fitness values for the pH 6.0→10.0→6.0 and pH 10.0→6.0→10.0 schemes were analysed separately instead of being pooled. Competitions with pH 6.0→10.0→6.0 and pH 10.0→6.0→10.0 schemes were also arranged between the two ancestors (unevolved *V. fischeri* EM17 and EM17Tn7) as controls (Fig. 2b). Finally, relative fitness values for all the derived lines were determined in FLM pH 7.4 to control for general lab adaptation. For these experiments, the derived lines and the ancestors were first acclimated in FLM pH 7.4 before being competed against one another (Fig. 2).

The relative fitness values among the acid specialists, alkaline specialists, and the pH generalists were dissimilar. As a result, there was a desire to compare relative fitness values from the FLM pH 6.0/10.0 derived lines to those of the FLM pH 6.0 and pH 10.0. Rather than use pooled Welch-Satterthwaite *t*-tests (for unequal sample sizes) to construct unplanned (*post hoc* or *a posteriori*) hypotheses with preexisting results, the above relative fitness assays were repeated to obtain independent data sets for the FLM pH 6.0, 10.0, and 6.0/10.0 derived lines [37, 42]. That is, these derived lines were compared to each other in how much better they were than the ancestors in the FLM pH environments where evolution took place. Hence, the derived lines were not competed directly against one another. Independent two-sample (unpaired) *t*-tests were used to analyse these results in a two-tailed manner [Type 1 α error=0.05; 37, 42]. For the FLM pH 6.0/10.0 derived lines, half of the competitions with the oppositely marked (CAMS/CAMR) ancestor were pH 6.0→10.0→6.0 and half were pH 10.0→6.0→10.0. These two schemes were then combined to make *n*=20.

### Animal experiments

Most experiments conducted with squid use unbuffered artificial seawater, which typically ranges between pH 7.7–8.3 [9]. However, artificial seawater buffered precisely at pH 8.0 was desired for the current study. For artificial seawater, pH 8.0 was selected for three main reasons. First, pH 8.0 is the midpoint (median) of the pH gradient examined in FLM pH 6.0–10.0. Second, pH 8.0 is close to the annual mean pH of the ocean [pH ~8.1; www.noaa.gov] [43]. Third, pH 8.0 is well within the optimal range for the squid *E. scolopes*. Much thought went into the selection of an appropriate buffer, since chemical toxicity to the squid was a concern. There were a number of possibilities. Previously, Tris (for pH 7.0–9.0) has been reported to be nontoxic to live shrimp, fishes, and molluscs [44–46]. Glycylglycine (for pH 7.5–8.9) has been found to be nontoxic to marine invertebrates, including echinoderms [47]. The inorganic buffers carbonate (for pH 6.0–8.0) and phosphate (for pH 5.8–8.0) are naturally present in the ocean. As a result, these definitely would be nontoxic for squid. For the present study, modified Sorensen phosphate buffer (final concentration for pH 8.0: 0.7 % w/v Na_2_HPO_4_ and 0.3 % w/v NaH_2_PO_4_) was selected over carbonate since phosphate is more stable [36]. Buffer mixture (10 mM MES, 10 mM HEPES, 10 mM EPPS, 10 mM CHES, and 10 mM CAPS) was also used for animal experiments. Pilot experiments conducted beforehand demonstrated buffer mixture was not toxic to squid hatchlings in any obvious way.

**Fig. 3.**
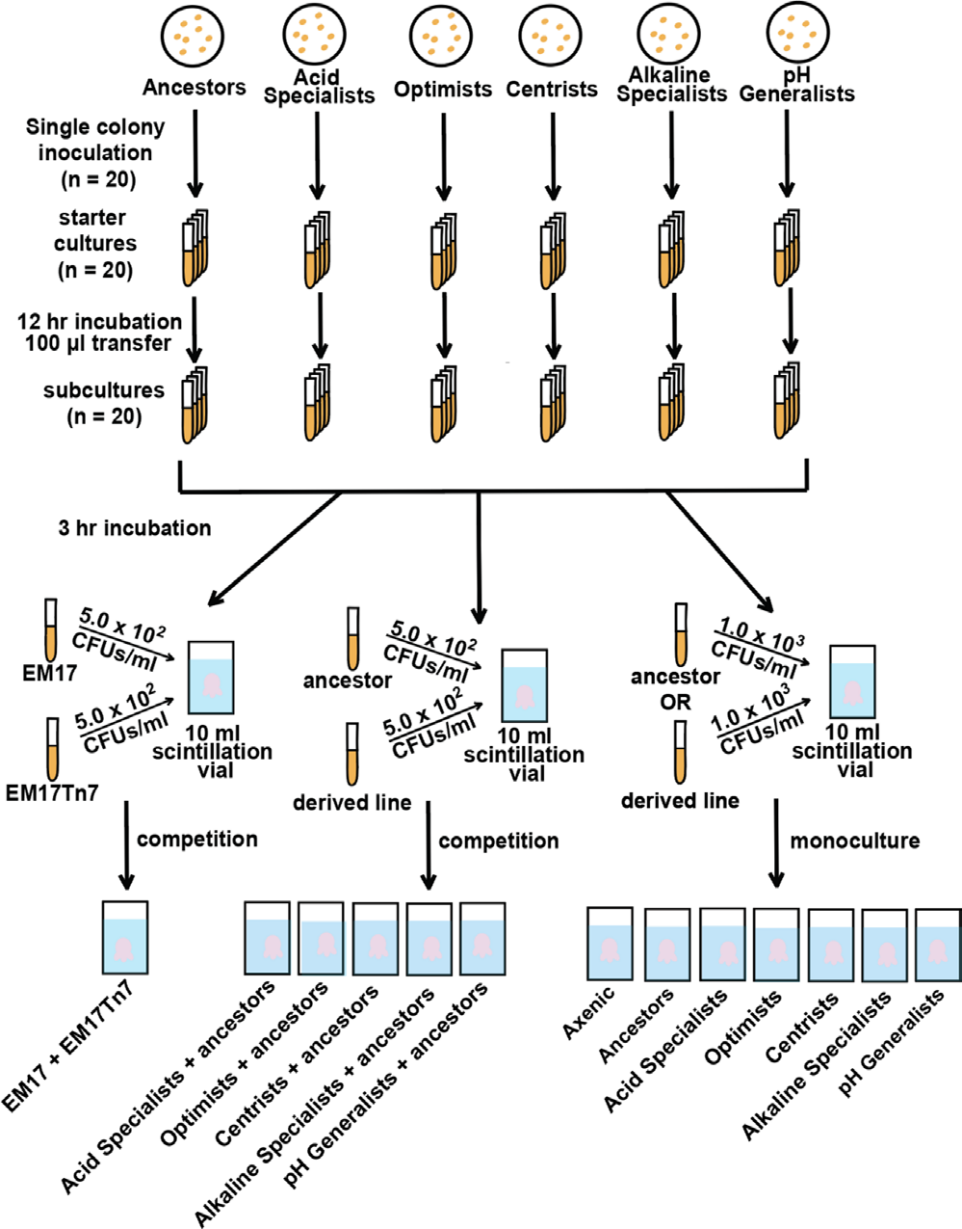
The *V. fischeri* ancestors and derived lines were grown and used to inoculate the squid hosts for monocultural and competition studies. Bioluminescence data was collected from the monoculture studies. Axenic (uninoculated) animals served as negative controls.

Using Instant Ocean (SS15-10), two batches of 34.0 ppt artificial seawater were made with double-distilled water [9]. One batch of artificial seawater was adjusted to pH 8.0 using modified Sorensen phosphate buffer, while another batch was adjusted to pH 8.0 with buffer mixture. These two batches of artificial seawater were called ASWP and ASWM, respectively. A refractometer was used to confirm the final salinities of ASWP and ASWM. For the animal experiments, the ancestors and derived lines were grown up as described earlier (Fig. 3). Monoculture experiments were set up, where squid were inoculated with only one lineage of bacteria, either a derived line or an ancestor but not both. For the monoculture experiments in squid, the log phase serial transfers for each derived line or ancestor were used to inoculate replicate 10 ml scintillation vials, with either 5.0 ml ASWP or ASWM (*n*=20), at a cell density of 1.0×10^3^ c.f.u.s ml^−1^. For the derived lines (*n*=20), each of the twenty replicates went into a single scintillation vial (Fig. 3).

Competition experiments were also set up, where squid were inoculated with a 1 : 1 ratio between a derived line and an ancestor (Fig. 3). Competitions were arranged between a derived line and an ancestor that were oppositely marked. For instance, EM2000 (CAMS) acid specialist was competed against ancestor *V. fischeri* EM17Tn7 (CAMR) in the squid (Table 1). Similarly for the cross experiment, TN2000 (CAMR) acid specialist was competed against ancestor *V. fischeri* EM17 (CAMS) in the squid. For the competition experiments in squid, the log phase serial transfers for each derived line and ancestor were used to co-inoculate replicate 10 ml scintillation vials with either 5.0 ml ASWP or ASWM (*n*=20). Co-inoculations between a derived line and an oppositely marked ancestor (CAMS/CAMR) were at a 1 : 1 ratio in ASWP and ASWM, and the cell density for each contestant in a competition was 5.0×10^2^ c.f.u.s ml^–1^ (Fig. 3). Hence, the combined cell density with both contestants was 1.0×10^3^ c.f.u.s ml^–1^. Analogously, competitions were also set up between ancestor *V. fischeri* EM17 and ancestor *V. fischeri* EM17Tn7 in ASWP and ASWM (*n*=20), which served as controls. Hence, all competitions performed in squid were between a derived line and an ancestor or between two ancestors (Fig. 3). In the current study, no two derived lines were competed directly against one another.

*E. scolopes* hatchlings just emerging from their eggs and possessing axenic light organs were used for all experiments. *E. scolopes* hatchlings were placed in 10 ml scintillation vials with either 5.0 ml ASWP or ASWM that were previously inoculated with bacteria as outlined earlier (Fig. 3). With an initial cell density of 1.0×10^3^ c.f.u.s ml^–1^ in the scintillation vials, squid hatchlings are guaranteed to be colonized by *V. fischeri* [9]. After a 3 h incubation with *V. fischeri *, animals were rinsed three times with ASWP or ASWM to synchronize symbiont colonization within this time window. Rinsed animals were then placed back into new 10 ml scintillation vials with either fresh 5.0 ml ASWP or ASWM. To create negative controls (*n*=20), animals were also placed in ASWP or ASWM (i.e. containing no *V. fischeri *) for 3 h before being rinsed (Fig. 3). Negative control animals were then put back into new 10 ml scintillation vials with either fresh 5.0 ml ASWP or ASWM. As a result, negative control squid were never exposed to *V. fischeri *. In summary, *n*=20 for all treatments and controls. Each replicate of the derived lines was inoculated into one squid animal for the monoculture experiments. Similarly for the competition experiments, each replicate of the derived lines was competed against an ancestor in one squid individual (Fig. 3).

All animals were maintained in a 12 h:12 h dark-light cycle at 25 °C. Fresh ASWP or ASWM was changed every 12 h [9]. At timepoint 48 h, squid hatchlings were placed in a Promega GloMax 20/20 luminometer to measure bioluminescence (as relative light units=RLUs). After this, animals were rinsed three times with either ASWP or ASWM. Squid were then sacrificed and their light organs homogenized in 1.5 ml microfuge tubes containing either ASWP or ASWM [9]. Teflon microfuge pestles were used to homogenize light organs. Light organ homogenates were serially diluted with either ASWP or ASWM serving as diluent. Hence, there were two sets of light organ homogenates, one in ASWP and another in ASWM [9]. The ASWP serial dilutions were spread onto FLP pH 8.0 agar plates with and without chloramphenicol, while those in ASWM were spread onto FLM pH 8.0 agar plates with and without chloramphenicol. As a precaution, both the ASWP and ASWM serial dilutions were also spread onto FLP pH 7.5 and FLM pH 7.5 agar plates with and without chloramphenicol, respectively. These plate counts were used to determine *V. fischeri* colonization levels in the squid host (CFUs/squid). Plate cultures were incubated for 24–48 h at 28 °C [9]. Animal experiments were statistically analysed using the least significant difference (LSD) procedure with a modified Bonferroni correction using the Dunn-Sidak method (experimentwise Type 1 α error=0.05) for all possible pairwise comparisons. The LSD procedure and Dunn-Sidak method are explained in great detail elsewhere [33, 37]. If the LSD error bars do not overlap, then the pairwise comparisons are significantly different.

### Correlational studies

Linear regression studies were implemented to demonstrate correlations between *V. fischeri* adaptation to pH stress and symbiosis with the sepiolid squid host. To this end, ‘Squid host colonization’ and ‘bioluminescence’ were each separately regressed onto relative fitness to pH stress (i.e. ‘adaptation to pH stress’) at 0, 400, 800, 1200, 1600, and 2000 generations. (See ‘Relative Fitness Assays’ and ‘Animal Experiments’ for methodological details.) Separate linear regressions were done for the *V. fischeri* EM and TN varieties at each pH stress, namely FLM pH 6.0, 10.0, and 6.0/10. These correlations were then statistically analysed as Model I linear regressions [37].

## RESULTS

### Lower and upper pH limits of growth for *V. fischeri* EM17 and EM17Tn7

**Fig. 4.**
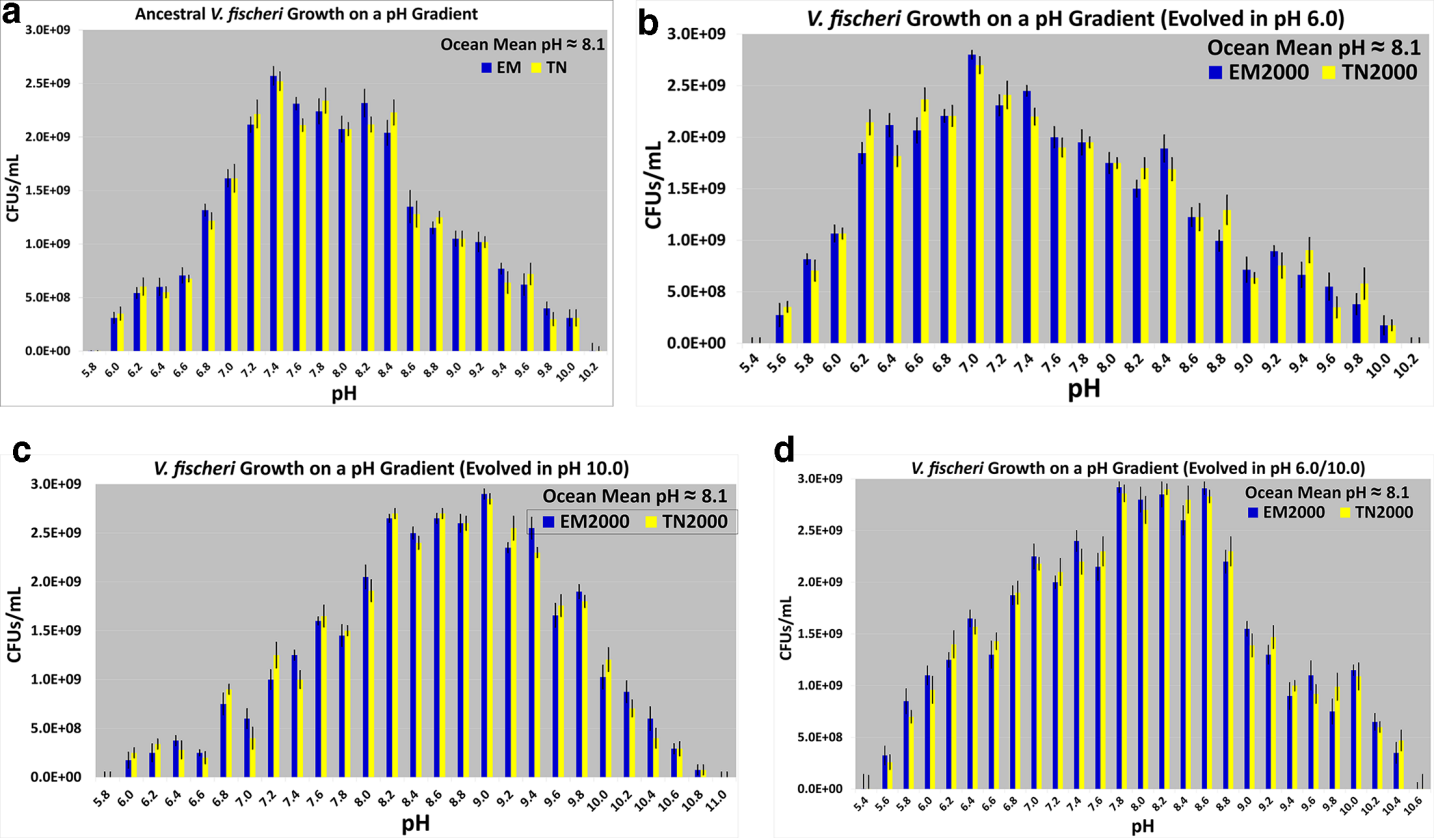
Growth distributions of the ancestors (4a.), acid specialists (4b.), alkaline specialists (4c.), and pH generalists (4d.) along a pH gradient (*n*=20 per pH). Error bars represent standard error of the mean.

To determine its pH niche space, *V. fischeri* was incubated over the entire pH range it was capable of growing. This permitted a rigorous assessment of what pH values were benign, optimal, and stressful to *V. fischeri *. This approach revealed what pH magnitudes were appropriate as ‘stressful challenges’ and benign controls for microbial experimental evolution with *V. fischeri *. The lower and upper pH limits of growth for the *V. fischeri* ancestors were pH 6.0 and pH 10.0 (Fig. 4a), respectively. The ancestors *V. fischeri* EM17 and EM17Tn7 grew along the entire pH gradient in FLS pH 7.0–9.0 (data not shown). As a result, the use of a medium with a buffer mixture (FLM) became necessary. There is no single buffer known that can cover the full pH spectrum that *V. fischeri* was able to grow in [34, 36]. The buffer with the widest known pH range is BTP (BIS-Tris-propane for pH 6.3–9.5), which would not have been useful for the current study [34]. The results reported here were from plate counts on FLM pH 7.5 agar. Fig. 4a shows the growth of the ancestors (unevolved *V. fischeri* EM17 and EM17Tn7) along a pH gradient in FLM broth. *V. fischeri* EM17 and EM17Tn7 displayed highly similar results. This is consistent with previous reports. Chloramphenicol resistance is known to be a neutral marker in *V. fischeri* [9, 32, 33]. The lower and upper growth limits were pH 6.0 and pH 10.0, respectively. No reproducible microbial growth occurred below or above these pH values. The mode, median, and mean of the distributions for *V. fischeri* EM17 in Fig. 4a were pH 7.4, 8.0, and 7.9, respectively. These values were identical for *V. fischeri* EM17Tn7.

Within the range pH 6.0–10.0, *V. fischeri* EM17 and EM17Tn7 were always able to at least grow to a cell density of 2.0×10^8^ c.f.u.s ml^–1^. In other words, microbial growth always at least occurred to 2.0×10^8^ c.f.u.s ml^–1^ or not at all. Apparently, even at extreme pH in FLM, there was at least moderate growth or none at all. This was a surprising result. Presumably, extreme pH values with maximal cell densities occurring at~1.0×10^7^ c.f.u.s ml^–1^ would have been expected. This result greatly facilitated the current microbial selection study. There was no worry about the extinction of evolving populations due to 100-fold dilutions by serially transferring into fresh media every 12 h [31, 48].

These results show that FLM pH 6.0 and pH 10.0 were the appropriate regimes to evolutionarily select for increased tolerance to extreme pH for 2000 generations in *V. fischeri *. The number of generations was determined through standard growth kinetics [3, 38]. Of course, a selection regime to the ‘optimal’ pH for growth is an obvious ‘nonstress’ control. FLM pH 8.0 is a natural choice, since it is the midpoint and approximate pH of ocean surface water [pH ~8.1; www.noaa.gov] [43]. However, FLM pH 7.4 was where maximal microbial growth occurred (Fig. 4a). This was an unanticipated result. Nonetheless, a similar result has been reported previously [26]. Rather than choose one over the other, both FLM pH 7.4 and 8.0 were used as ‘nonstress’ controls. Similar results were obtained with plate counts on FLM agar at other pH values (data not shown).

### Growth of derived bacteria in pH gradient

**Fig. 5.**
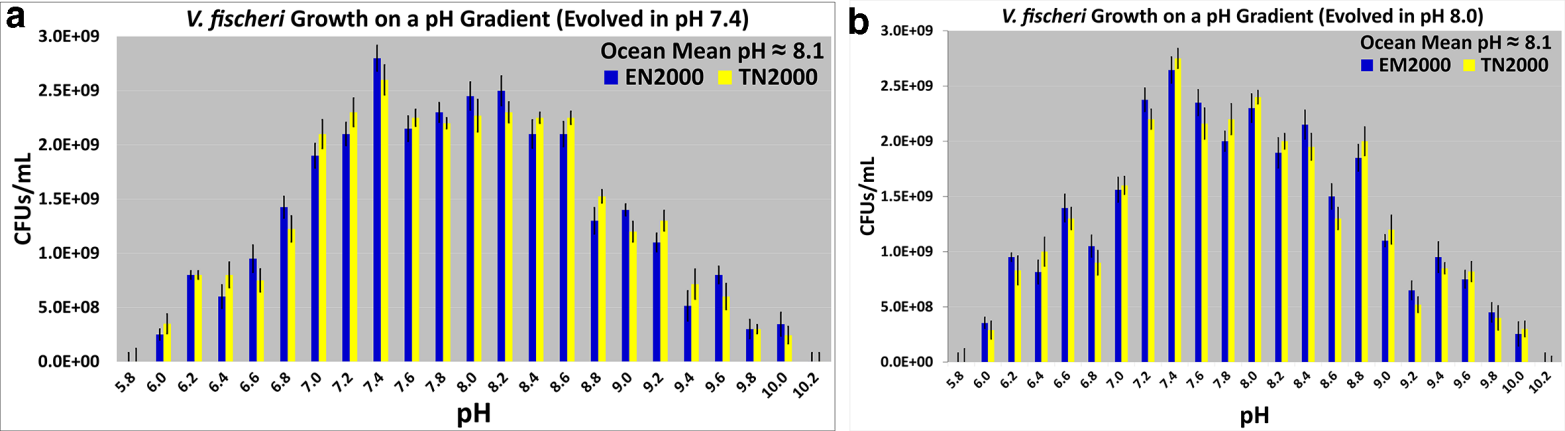
Growth distributions of optimists (5a.) and centrists (5b.) along a pH gradient (*n*=20 per pH). Error bars represent standard error of the mean.

The results reported here were from plate counts on FLM pH 7.5 agar. Figs 4 and 5 illustrate how the acid specialists, optimists, centrists, alkaline specialists, and pH generalists grew in a pH gradient in FLM pH 5.4–11.0. The modes, medians, and means for these growth distributions are reported below. For each selection regime, the results were quite similar for the two derived (EM2000 and TN2000) varieties. For the acid specialists, the mode, the median, and mean changed relative to the ancestors. These values were now pH 7.0, 7.8, and 7.5, respectively (Fig. 4b). Fig. 4b is skewed to the right. The acid specialists still possessed pH 10.0 as the upper growth limit, but these lines did not grow to as high a cell density as the ancestors at pH 10.0 (Fig. 4a). Moreover, the acid specialists were able to grow in more acidic environments than the ancestors (down to pH 5.6, Fig. 4b).

The mode, the median, and mean for the alkaline specialists changed to pH 9.0, 8.4, and 8.6, respectively (Fig. 4c). The alkaline specialists still possessed pH 6.0 as a lower limit of growth, but these lines displayed a lower growth yield than the ancestors at pH 6.0. The alkaline specialists possessed a higher pH limit of growth than the ancestors (pH 10.8, Fig. 4c). Fig. 4c is skewed to the left. The growth distributions were unimodal for the ancestors (Fig. 4a). They were also unimodal for the acid and alkaline specialists. For the pH generalists, the growth distributions along the pH gradient are multimodal (Fig. 4d). For the pH generalists, the modes reside between pH 7.8 and 8.8. The median and mean of the growth distributions for the pH generalists are pH 8.0 and 7.9, respectively. The lower growth limit decreased relative to the ancestors (pH 5.6), while the upper one increased (pH 10.4). The growth distributions for the optimists and centrists have the same mode, median, and mean as the ancestors (Fig. 5a,b). Similar results were obtained with plate counts on FLM agar at other pH values (data not shown).

### Relative fitness values

**Fig. 6.**
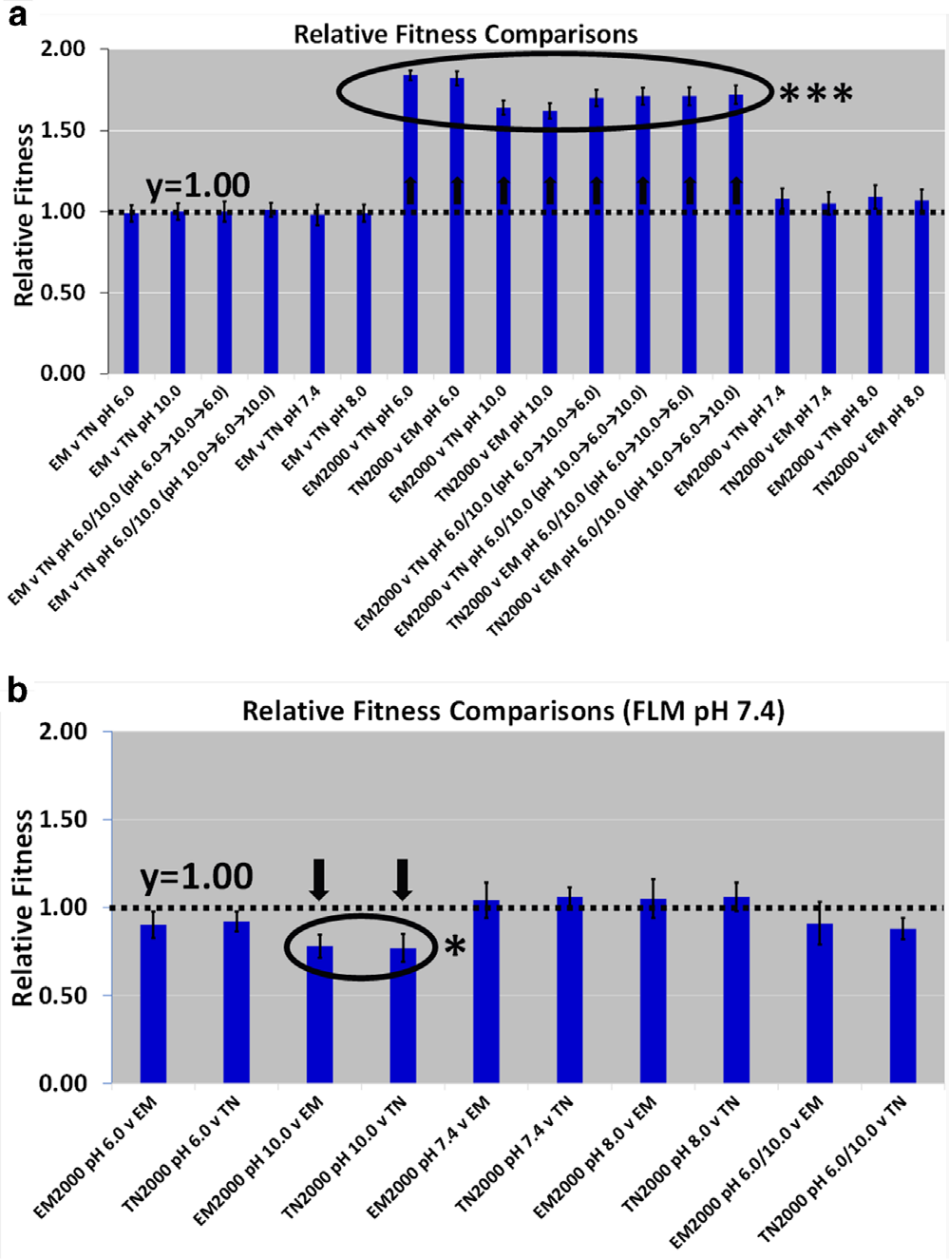
Relative fitness values were calculated with one-sample *t*-tests (two-tailed, Type 1 α error=0.05) and compared to *H*_0_=1.00 (*n*=20). Relative fitness of the evolved lines were competed against the ancestors after 2000 generations. 6a. Competitions occurred between ancestors and derived lines in FLM pH where evolution took place. 6b. Competitions occurred between ancestors and derived lines in FLM pH 7.4. Key: v=versus, (pH 6.0→10.0→6.0)/(pH 10.0→6.0→10.0)=competition scheme involving pH generalists, EM=ancestral/unevolved *V. fischeri* EM17, TN=ancestral/unevolved *V. fischeri* EM17Tn7, EM2000=* V. fischeri* EM17 evolved for 2000 generations, and TN2000=* V. fischeri* EM17Tn7 evolved for 2000 generations. Error bars represent standard error of the mean. The broken horizontal line signifies y=1. Circled means are significantly different (*=0.01 <*P*≤0.05 and ***=*P*≤0.001), and arrows indicate the direction that relative fitness changed.

Using the ‘frozen fossil record’ constructed during the course of microbial experimental evolution to the different pH regimes, the ancestral and derived lines were compared against one another at different evolutionary time points to assess relative fitness. The main aim here was to determine whether microbial adaptation occurred to benign, optimal, and stressful pH. Adaptation to benign and optimal pH were controls utilized to assess adaptation to pH values that were taxing to microbial growth. The ultimate goal was to determine whether microbial adaptation to pH stress, when compared to microbial evolution at benign and optimal pH, significantly affected *V. fischeri *’s symbiosis with the squid host (see ‘Animal Experiments’ in Results section). The results reported here were from plate counts on FLM pH 7.5 agar. Fig. 6a shows the data for the relative fitness assays between the ancestral and derived lines. Competitions were either between two ancestors or between an ancestor and a derived line that were oppositely marked (CAMS/CAMR). Competitions between an ancestor and a derived line took place only in the pH environment that the derived line evolved in. No competitions were between two derived lines. Additionally, no competitions were between two contestants possessing identical markers (e.g. no CAMR versus CAMR). Competitions between two ancestors (EM versus TN) were ‘neutral marker’ controls. In Fig. 6a, the derived line is always listed first in competitions between the ancestral and derived lines.

None of the relative fitness values for the ‘ancestor versus ancestor’ (EM v TN) experiments were significantly different from 1.00 in FLM pH 6.0, 7.4, 8.0, 10.0, and 6.0/10.0 (Fig. 6a). This demonstrates CAMR is a neutral marker in all the pH selection regimes. *V. fischeri* successfully adapted to FLM pH 6.0, 10.0, and 6.0/10.0, as all of these relative fitness values were significantly larger than 1.00. *V. fischeri* evolution in pH 7.4 and 8.0 displayed a trend of being ~5–10 % greater in fitness relative to the ancestors. Nonetheless, these relative fitness increases in FLM pH 7.4 and 8.0 were not significantly different from 1.00. Apparently, there was no appreciable selection pressure for *V. fischeri* to adapt to benign pH values within 2000 generations. Fig. 6a indicates *V. fischeri* EM17 and EM17Tn7 both similarly responded to the selection pressures of FLM pH media. In summary, there was at least a ~80, ~60, and ~70 % increase in relative fitness in FLM pH 6.0, 10.0, and 6.0/10.0, respectively. These increases were significantly different. There were no fitness changes in FLM pH 7.4 and pH 8.0 that were significantly different. The pH 6.0/10.0 relative fitness values appear similar for the two acclimation schemes (Fig. 6a). Fig. 6b shows the relative fitness values for the derived lines in FLM pH 7.4, when they are compared to the ancestors. Only the alkaline specialists possess relative fitness values that were significantly different (about ~20 % lower) from the ancestors in FLM pH 7.4. These reduced fitness changes were due to tradeoffs in FLM pH 7.4 that resulted from adaptation to FLM pH 10.0. Compare microbial growth in FLM pH 7.4 in Fig. 4a,c. The alkaline specialists struggle to reach a cell density of ~1.0×10^9^ c.f.u.s ml^–1^, while all the other derived lines and the ancestors can reach a cell density of ~2.0×10^9^.

### Specialists and generalists

The relative fitness values of the acid specialists, alkaline specialists, and pH generalists were all different from one another (Fig. 6a), yet they were all significantly more fit than the ancestors in the stressful pH environments where adaptation had occurred. This raised the question whether there were any significant differences among the specialists and generalists in how much they adapted to their stressful pH regimes. In other words, which adapted more to their stressful pH environments, specialists or generalists? The results reported here were from plate counts on FLM pH 7.5 agar. The *V. fischeri* EM17 and EM17Tn7 lines that underwent 2000 generations of evolution in FLM pH 6.0, 10.0, and 6.0/10.0 were once again competed against their oppositely marked (CAMS/CAMR) ancestor to determine relative fitness. Independent (unpaired) two-sample *t*-tests were conducted between these competitions (two-tailed, Type 1 α error=0.05). Separate experiments were conducted for each *t*-test comparison, and the data set in Table 2 is completely independent of the one in Fig. 6a. That is, the *t*-test contrasts in Table 2 are not *post hoc* pairwise comparisons of data in Fig. 6a. Relative to the ancestors, Table 2 shows that acid specialists can improve their fitness values (~80–90 %) in pH 6.0 more than alkaline specialists can improve their fitness (~60 %) in pH 10.0. Relative to ancestors, the amount of adaptation by the specialists was not significantly different from the pH generalists. The pH generalists improved their fitness by ~70–80 % in temporally fluctuating FLM pH 6.0/10.0, when compared to ancestral lines. All *t*-test comparisons were within varieties and not between. Moreover, there was no evidence that two varieties ever responded to a pH selection regime differently. For example, EM2000 pH 10.0 RF and TN2000 pH 10.0 RF in Table 2 were similar. Similar results were obtained with plate counts on FLM agar at other pH values (data not shown).

**Table 2.** *
V. fischeri* EM17 and EM17Tn7 evolved in FLM pH 6.0, 10.0, and 6.0/10.0 were competed against their opposingly marked ancestor (*n*=20). Independent two-sample *t*-tests were conducted between these competitions (two-tailed, Type 1 α error=0.05) The same key in Fig. 1 is also used here for ‘EM2000’, ‘TN2000’, etc.

Independent Comparison	RF Mean 1 (±SE) v RF Mean 2 (±SE)
EM2000 pH 6.0 RF v EM2000 pH 10.0 RF	1.84 (±0.074) v 1.61 (±0.062)*
TN2000 pH 6.0 RF v TN2000 pH 10.0 RF	1.92 (±0.081) v 1.58 (±0.089)**
EM2000 pH 6.0 RF v EM2000 pH 6.0/10.0 RF	1.85 (±0.058) v 1.73 (±0.049) ns
TN2000 pH 6.0 RF v TN2000 pH 6.0/10.0 RF	1.86 (±0.050) v 1.79 (±0.061) ns
EM2000 pH 10.0 RF v EM2000 pH 6.0/10.0 RF	1.60 (±0.054) v 1.69 (±0.059) ns
TN2000 pH 10.0 RF v TN2000 pH 6.0/10.0 RF	1.59 (±0.061) v 1.68 (±0.069) ns

ns=*P*>0.05 (not significant).

*=0.01 <*P*≤0.05, **=0.001 <*P*≤0.01.

### Animal experiments data

The hypothesis being tested here was whether a microorganism (* V. fischeri *) experiencing evolution to an environmental stressor (pH) during the free-living stage affected the microbe’s ability to engage in symbiosis with its host (the Hawaiian bobtail squid *E. scolopes*). Herein, there were two parameters that represented measures of ‘symbiosis’. The first was squid host colonization. The second was bioluminescence induced in the squid host. Previous work with microbial experimental evolution in the squid-* Vibrio* mutualism has shown that these parameters are appropriate measures or readouts of ‘symbiosis’ [9, 18, 28]. In this study, *E. scolopes* were inoculated with the ancestral and derived *V. fischeri* lines in monoculture and in 50 : 50 competitions. Host colonization and squid bioluminescence were then monitored. *V. fischeri* lines evolved in benign and optimal pH served as controls for the ‘stress’ treatments. Animals never inoculated with any *V. fischeri* (i.e. light organs remaining in the axenic or gnotobiotic condition) were used as negative controls.

**Fig. 7.**
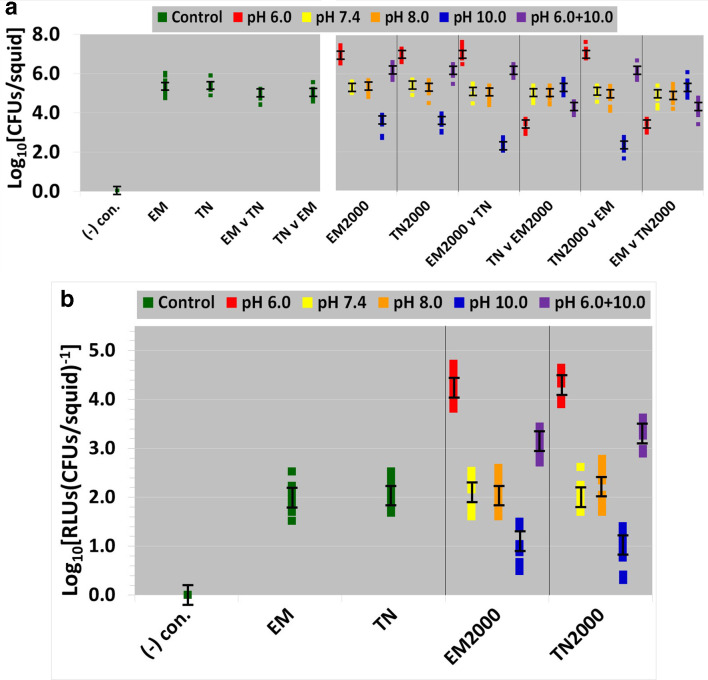
Squid hatchlings were inoculated with *V. fischeri *. 7a. The squid monocultural and competition data are shown with the ancestral and derived lines. Monocultures only list one participant at the bottom of the diagram, while the competitions list both contestants. For the competitions, the colonization level (log_10_[CFUs/squid]) shown is for the first contestant of a pair. The second contestant of a pair is the opponent for that particular competition. 7b. Bioluminescence data of squid monocultures (pure cultures). For (a) and (b), the different colors represent either axenic animals (negative control), ancestors (positive controls), or the derived lines (in monoculture and competition). Squares represent individual data points. Key: (-) neg.=negative control, v=versus, EM=ancestral/unevolved *V. fischeri* EM, TN=ancestral/unevolved *V. fischeri* TN, EM2000=* V. fischeri* EM evolved for 2000 generations, and TN2000=* V. fischeri* TN evolved for 2000 generations. There are no competitions in (b). For (a) and (b), error bars represent least significant difference (LSD) of the mean.

The results reported here were from plate counts on FLM pH 7.5 agar. Fig. 7a shows the squid colonization data in ASWP pH 8.0. Error bars in Fig. 7a signify the LSD for this entire data set. Means with overlapping LSD error bars are not significantly different [37]. For the monocultural squid colonization experiments (hatchlings inoculated in pure culture), only a single participating strain is listed at the bottom of Fig. 7a. The competition studies in the squid are also shown in Fig. 7a. Participants in each competition are always oppositely marked (CAMS/CAMR). In the squid, competitions were either ‘ancestor versus ancestor’ or ‘ancestor versus derived line’. There were no ‘derived line versus derived line’ competitions. At the bottom of Fig. 7a, for the squid competition experiments, both of the contestants are listed that were co-inoculated in a 1 : 1 ratio with the animals. For these competitions in the animal hosts, the amount of squid colonization is listed for the first member of a pair of contestants. The second member of a pair represents the opponent for that particular competition. Negative control animals were not colonized. There were no significant differences in the squid colonization levels between the ancestors *V. fischeri* EM17 and EM17Tn7 in monoculture or competition. Both colonize squid equally. CAMS/CAMR was a neutral marker for squid colonization (Fig. 7a).

The highest level of squid colonization in monoculture was accomplished by acid specialists, while the lowest level of monocultural colonization was by alkaline specialists. Both of these squid colonization levels were significantly different from the ancestors (Fig. 7a). The pH generalists were intermediate to the acid and alkaline specialists in monocultural squid colonization, yet the pH generalists still possessed more monocultural colonization than the ancestors that was significantly different. Both the optimists and centrists were not significantly different from the ancestors for squid colonization in monoculture (Fig. 7a). Acid specialists outperformed the ancestral lines in the squid competitions, where both contestants were co-inoculated at a 1 : 1 ratio with the animals. Alkaline specialists were surpassed by the ancestral lines in the squid competitions. The pH generalists also outpaced the ancestors in the squid competitions. Neither optimists nor centrists were significantly different from the ancestors in the squid competitions. Consequently, evolution in the benign part of the pH continuum (pH 7.4 and 8.0) had no effect on *V. fischeri *’s ability to colonize squid. To summarize, the monocultural colonization levels in the squid possessed a transitive relationship: acid specialists>pH generalists>ancestors=optimists=centrists>alkaline specialists>axenic animals. The squid competitions generally mirrored this relationship.

Fig. 7b shows the bioluminescence data only for the squid monocultures in ASWP pH 8.0. The bioluminescence data is omitted for the squid competitions, since there is no way to attribute bioluminescence in an animal to a single strain or line. Error bars in Fig. 7b represent the LSD for this whole data set. Means with overlapping LSD error bars are not significantly different. Negative control animals were not bioluminescent. The squid hosts colonized by the two ancestors (EM and TN) were not significantly different from each other in bioluminescence levels, demonstrating the neutrality of CAMS/CAMR. Squid hatchlings colonized by acid specialists were significantly brighter than animals colonized by the ancestors (Fig. 7b). Animals colonized by alkaline specialists were significantly dimmer than those colonized by the ancestors. Squid colonized by the pH generalists were significantly brighter than animals colonized by either the ancestors or the alkaline specialists. However, squid colonized by the pH generalists were significantly dimmer than animals colonized by acid specialists (Fig. 7b). Bioluminescence levels in animals colonized by optimists and centrists were not significantly different from those colonized by the ancestors. In summary, the brightness levels in the squid hosts possessed a transitive relationship: acid specialists>pH generalists>ancestors=optimists=centrists>alkaline specialists>axenic animals. Similar ASWP animal data were obtained with plate counts on FLP and FLM agar at other pH values (data not shown). Additionally, similar animal results were obtained with ASWM pH 8.0 (data not shown). Thus, the squid colonization and bioluminescence data obtained from ASWP and ASWM were quite similar.

### Correlational studies

**Fig. 8.**
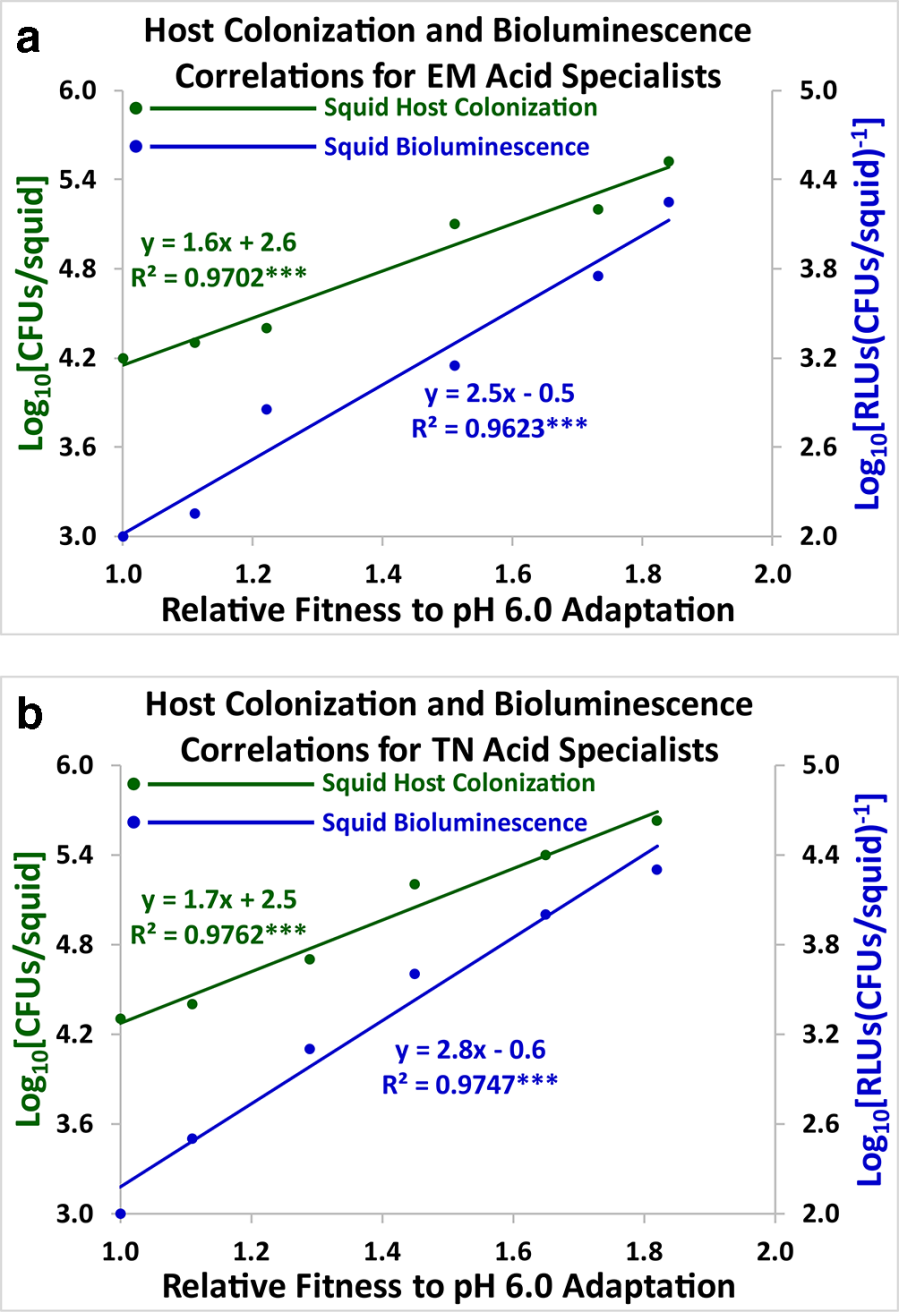
Linear correlations for the EM2000 (8a) and TN2000 (8b) varieties are shown for the acid specialists. Host colonization (green) and squid bioluminescence (blue) for *V. fischeri* are regressed against adaptation to the acidic growth limit (pH 6.0). Each data point represents the arithmetic mean of twenty lines (*n*=20) at 0, 400, 800, 1200, 1600, and 2000 generations. The slopes are positive and statistically significant.

**Fig. 9.**
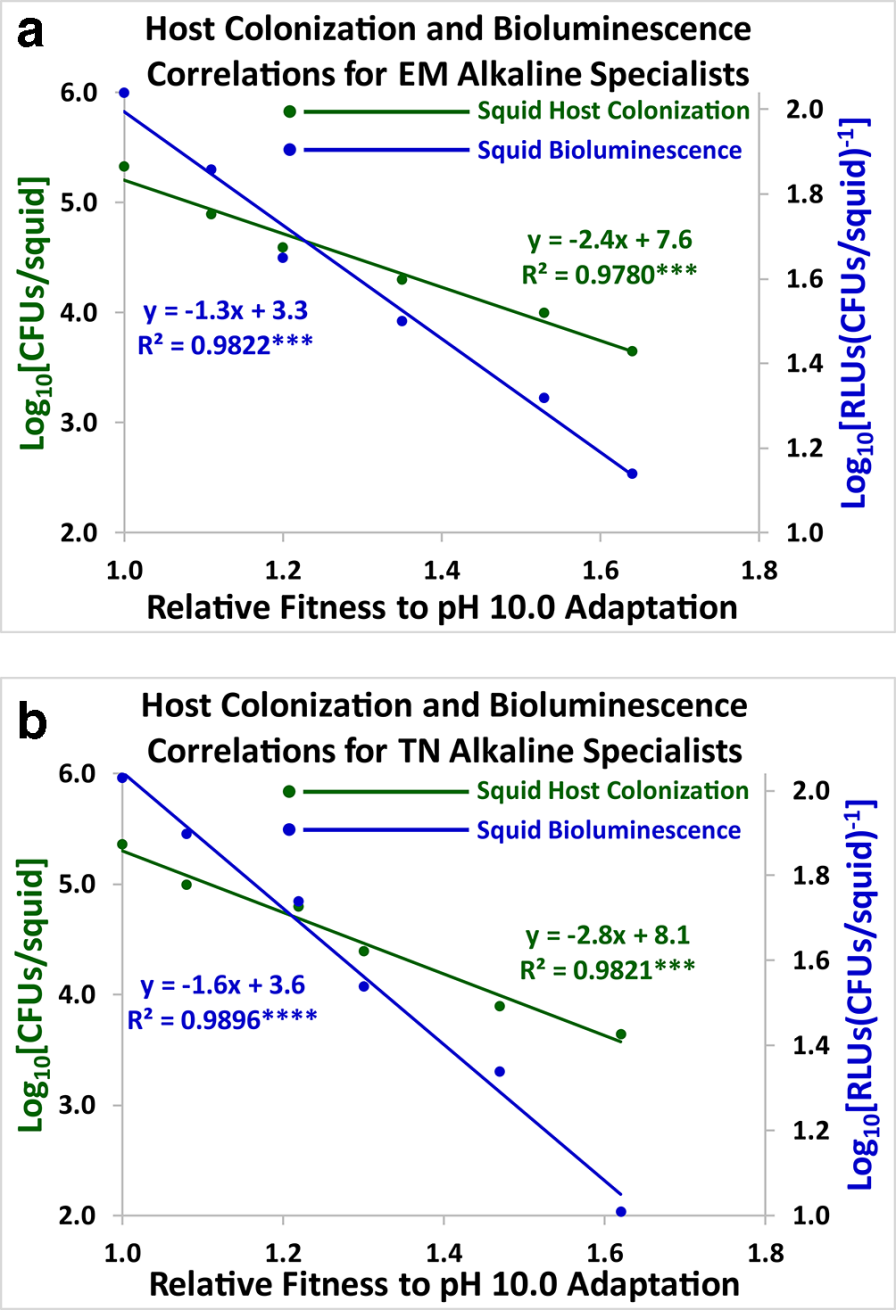
Linear correlations for the EM2000 (9a) and TN2000 (9b) varieties are shown for the alkaline specialists. Host colonization (green) and squid bioluminescence (blue) for *V. fischeri* are regressed against adaptation to the alkaline growth limit (pH 10.0). Each data point represents the arithmetic mean of twenty lines (*n*=20) at 0, 400, 800, 1200, 1600, and 2000 generations. The slopes are negative and statistically significant.

**Fig. 10.**
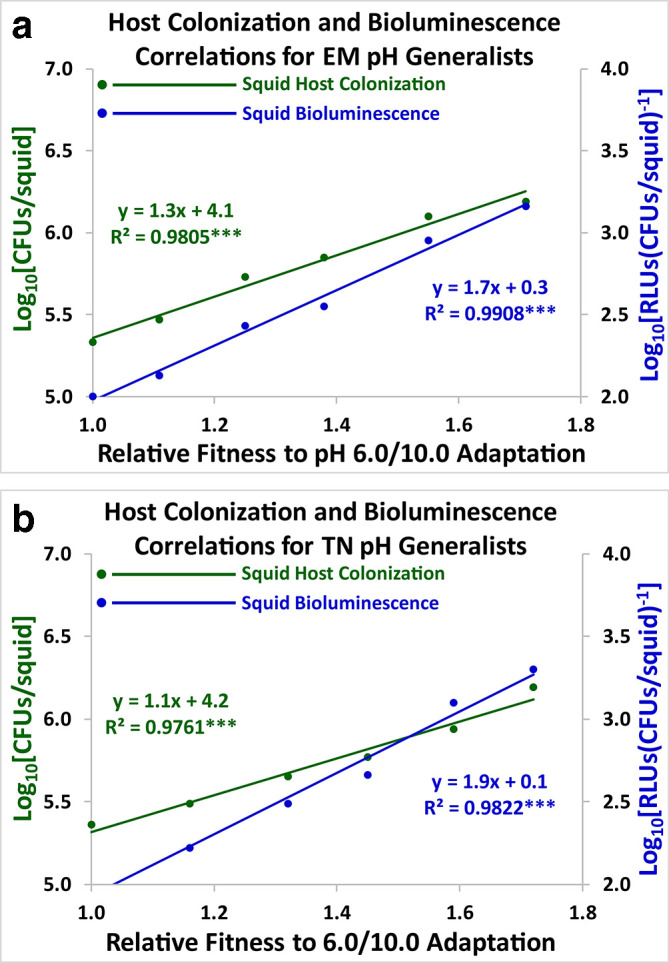
Linear correlations for the EM2000 (10a) and TN2000 (10b) varieties are shown for the pH generalists. Host colonization (green) and squid bioluminescence (blue) for *V. fischeri* are regressed against adaptation to fluctuation between the acidic and alkaline growth limits (pH 6.0/10.0). Each data point represents the arithmetic mean of twenty lines (*n*=20) at 0, 400, 800, 1200, 1600, and 2000 generations. The slopes are positive and statistically significant.

Host colonization (green) and squid bioluminescence (blue) were graphed against adaptation to pH stress (Figs 8–10). All the resulting scatter plots had slopes that were statistically significant using Model I linear regression analysis [37]. Positive linear correlations were demonstrated between both symbiosis parameters and adaptation to pH stress over the course of 2000 generations for the acid specialists and the pH generalists (Figs 8 and 10). Negative linear correlations were exhibited for the alkaline specialists (Fig. 9). Host colonization and squid bioluminescence steadily increase for the acid specialists and the pH generalists, while squid colonization and bioluminescence both descend for the alkaline specialists. Results were similar for both the ET2000 and TN2000 varieties. Hence, antibiotic resistance did not greatly impact the evolutionary trajectories of the acid specialists, the alkaline specialists, and the pH generalists.

## DISCUSSION

In this study, the pH lower and upper limits were determined to be pH 6.0 and pH 10.0 in FLM (Fig. 4a) for *V. fischeri *, which clearly represented acid and alkaline stress for this microorganism. *V. fischeri* successfully adapted to these extreme pH values and their fluctuations (Fig. 6a). Moreover, there was no significant adaptation to benign pH, which were pH 7.4 and 8.0. Relative to the ancestors, the ability of *V. fischeri* to grow along a pH gradient was greatly affected as a result of adaptation to stressful pH (Fig. 4b–d). Hence, the relative symmetry and kurtosis of the growth distribution along a pH gradient changed as a result of evolution to stressful pH. This was not true for evolution to benign pH 7.4 and 8.0 (Fig. 5a,b). *V. fischeri* was more able to increase its relative fitness to acid stress than alkaline stress, ~80 % versus ~60 %, respectively (Fig. 6a). This difference was significant (Table 2). However, neither of these quantities were significantly different from the improvement in relative fitness observed in temporally fluctuating acid and alkaline stress (~70 %). Only the alkaline specialists displayed a statistically significant tradeoff at pH 7.4 (ancestral pH optimum, Fig. 4a) as a result of adaptation to stressful pH (Fig. 6b). None of the other derived lines manifested a significantly different change at pH 7.4.

Indeed, pH stress evolution during the free-living phase impacted *V. fischeri *’s ability to colonize its squid host (Fig. 7a). *V. fischeri *’s capacity to induce bioluminescence in its host was also affected (Fig. 7b). Since evolution in benign pH 7.4 and 8.0 lacked these outcomes, *V. fischeri* adaptation to media components in FLM cannot be attributed as major contributing factors. Interestingly, pH stress evolution exhibited both facilitative and deleterious effects on *V. fischeri *’s ability to colonize its native animal host. More generally, this study demonstrates that microbial stress evolution during the free-living phase can have both positive and negative effects on host-microbe interactions. Model I linear regression analyses substantiate this claim (Figs 8–10). Although this result was shown for a mutualism, the current findings are applicable to commensalisms and pathogeneses (parasitisms) [5]. Since the transitive relationships observed in the bioluminescence data paralleled the squid colonization results, one outcome may be the direct consequence of another. Increased bioluminescence has been associated with enhanced squid host colonization in previous studies [9, 33]. Gene knockouts in bioluminescence or quorum sensing disrupt *V. fischeri *’s symbiosis with the squid host [49]. Adaptation to acid stress may enable *V. fischeri* to use more oxygen, while in the squid light organ, for increasing its bioluminescence [50].

Upon hatching, the squid light organ has a pH ~7.0–8.0 each day [51]. However, a transition occurs in the light organ between weeks 2 and 4 post-hatching. The light organ begins to adopt a daily cycle, where the pH environment declines throughout the day. By week 4, the light organ is pH ~7.0 immediately after venting at dawn. Over the next 24 h, the light organ pH gradually decreases to pH ~5.5 just prior to the next venting event that occurs at sunrise [51]. Symbiont catabolism of carbon substrates provided by the host leads to the accumulation of organic acids, which decreases the pH in the light organ. While no microbial growth occurred below pH 6.0 in the ancestral lines of *V. fischeri* (Fig. 4a), the derived lines that adapted to pH 6.0 and 6.0/10.0 (acid specialists and pH generalists) were able to grow down to pH 5.6 (Fig. 4b,d). (The alkaline specialists were also able increase their growth limit to pH 10.8, Fig. 4c.) Perhaps microbial adaptation to a high-nutrient environment containing acid stress allows growth at lower pH levels that were previously impermissible. Interestingly, the acid and alkaline specialists retained the same upper and lower pH limits of growth as the ancestors, respectively. Likewise, the pH generalists were able to expand the lower and upper limits of growth (Fig. 4d). The ‘transition’ period in the light organ may select for *V. fischeri* that will eventually be able to grow amid acid stress [51].

Another reason why *V. fischeri* might be able to grow at pH ~5.5 in the squid light organ but not in FLM pH 5.6 has to do with K^+^ cations. Microorganisms can use K^+^ cations to maintain homeostasis against pH stress [52, 53]. The K^+^ concentration in FLM is similar to natural seawater [4]. The K^+^ concentration is likely to be much higher in the squid light organ relative to ambient seawater. Within the light organ, *V. fischeri* resides in extracellular spaces called crypts, which are anatomically analogous to lumina [54]. The eukaryotic host cells comprising the light organ possess an intracellular K^+^ concentration that is at least 10–100 times greater than in seawater [55]. A residual amount of host cells (crypt epithelial cells and hemocytes) in the light organ lyse throughout the day, which releases nutrients to *V. fischeri *. Host cells that surround *V. fischeri* in the crypt spaces are also capable of secreting nutrients [51, 56]. Hence, within the squid light organ, different modes exist that could supplement extra K^+^ cations to *V. fischeri *.

The selection regimes pH 6.0, 10.0, and 6.0/10.0 represent realistic stressful magnitudes *V. fischeri* could encounter in nature. Supporting Information details the extreme pH values and drastic pH fluctuations that *V. fischeri *, Vibrionaceae, and other marine microorganisms are likely to encounter in aquatic environments. For example, the seasonal maximum approaches pH ~10.0 at Mariager Fiord in Denmark, where the organic nutrient load is high and circulation exchange with the open ocean is constrained [57]. At the other extreme, a pH~6.0 is not uncommon in marine waters, where there is extensive upwelling of inorganic nutrients, high influx of dissolved organic carbon, and active geological activity [58, 59]. Thus, extreme pH in aquatic environments are frequently associated with high-nutrient environments, which justifies the use of FLM pH medium for the pH selection regimes in this study. The use of *V. fischeri* was also ideal for this study, since the Vibrionaceae are adept and proficient at initiating host-microbe interactions with eukaryotic hosts, including mutualisms, commensalisms, and pathogeneses [21, 27]. *V. fischeri* EM17 itself would not have experienced in nature the stressful pH values used in the current study. This strain was isolated from Japanese coastal waters, where the pH does not fluctuate to the extremes investigated herein [60].

The harsh pH environments that can be found in aquatic habitats for the present day is characterized in Supporting Information. There is much lively discussion about the pH environments of the early oceans that formed during the Archean Eon (3.85–2.50 billion years ago) and Proterozoic Eon (2.50–0.54 billion years ago) [61, 62]. One view is the Precambrian ocean was acidic owing to the volcanic gases (CO_2_, H_2_S, SO_2_, NO_2_, and hydrogen halides) that might have been present in the early atmosphere of the Earth [63]. For an acidic Archean ocean, possible values of pH 5.0 to 5.7 have been reported [61, 64]. In contrast, the ‘soda ocean’ hypothesis argues for an Archean ocean that was alkaline at pH 9.0 to 11.0 [65]. Another view is that seafloor weathering (the carbonate–silicate geochemical cycle) has always buffered the ocean pH to the near-current value, permitting only modest vicissitude of ±0.5 pH units throughout Earth history [61, 66]. A circumneutral pH (pH ~7.0) for the Archean ocean has also been proposed as a result of rare element anomalies observed in the geological record [61]. Despite these divergent perceptions regarding the hydrogen ion concentration of the Archean ocean, many researchers believe that the annual mean pH of the ancient sea surface approached a value within the range of pH 7.5–8.5 by the late Proterozoic Eon [67, 68]. Since the Cambrian Period (541–55.6 million years ago) until modern times (i.e. Phanerozoic Eon), the ocean pH fluxes have largely been constrained between pH 7.4 and pH 9.0 [68, 69]. Vibrionaceae are within the phylum Proteobacteria, which first arose 2.0–3.0 billion years ago based on molecular clock calculations, phylogenomic dating, and considerations of the geologic time scale [70–72]. Thus, Vibrionaceae ancestry experienced the pH fluxes that were present in the oceans of the Archean and Proterozoic Eons [73].

Eukaryotes are estimated to have originated 2.0–2.7 billion years ago, which means eukaryotic natural history largely overlaps with the evolution of Proteobacteria [73–75]. The phylum Proteobacteria includes many taxonomic groups (e.g. Vibrionaceae, Rhizobiaceae, and Rickettsiaceae) with a high propensity for entering into host-microbe interactions [76]. The ancestor of mitochondria, undergoing endosymbiosis with eukaryotes at least 2.0 billion years ago, belongs to the Proteobacteria lineage [77]. Proteobacteria and early eukaryotic microbes may have engaged in host-microbe associations at a time, when ancient oceans were either highly acidic or alkaline. Hence, extreme pH may have actively influenced initial events in the coevolution between Proteobacteria and eukaryotes, including symbiogenesis [78]. Today there are known examples of Proteobacteria, including *Vibrio *, that have cyclical free-living and intracellular host-associated stages with eukaryotic microbes [79]. More generally, environmental stressors—temperature, salinity, and ultraviolet light for example—may have played a much larger role than previously appreciated in shaping the evolution of host-microbe relationships through geological time.

To summarize, many microorganisms involved in host-microbe interactions possess life histories that contain cyclical free-living and host-associated stages [28]. This study demonstrated that stressful selection pressures during the free-living stage can influence host-microbe interactions. Microbial evolution to stressful pH had both facilitating and deleterious effects on the ability of *V. fischeri* to colonize its squid host. In prior work, biofilm evolution during the free-living phase was shown to improve the ability of *V. fischeri* to infect their squid hosts [28]. Numerous opportunities exist in marine environments for microorganisms to be subjected to positive selection pressure for increased biofilm formation, including sediment, suspended particulate matter (e.g. colloids), and marine snow [80]. Other circumstances favoring the evolution of biofilm formation include the seabed and its geological features (e.g. underwater lava pillars). Hydrothermal vents, cold seeps, and stromatolites are particularly well studied systems. Incidentally, niches with severely low (<pH 4) and high pH (>pH 10) can be found in hydrothermal vents [81]. Thus, hydrothermal vents are localities which potentially subject marine microbes to pH stress and heavy selection pressure for elevated biofilm development. *Vibrio* species have been isolated from hydrothermal vents, *V. diabolicus* and *V. antiquaries* for instance [82, 83]. Vibrios from hydrothermal vents frequently form intimate associations with animal hosts, including annelid worms. In conclusion, microbial adaptations acquired during the free-living phase that enable higher stress resistance and enhanced biofilm formation may augment a microorganism’s proficiency to establish relationships with eukaryotic hosts. An elevated propensity by a microbe to form biofilms is itself correlated with a higher ability to tolerate environmental stressors, including pollutants, toxins, and antibiotics [84]. Further research into this field is necessary in light of growing concerns over climate change and ocean acidification.

## Supplementary Data

Supplementary material 1
