# Supplementary material for: Adaptation to pH stress by Vibrio fischeri can affect its symbiosis with the Hawaiian bobtail squid (Euprymna scolopes)
Source: Microbiology (Reading). 2020 Jan 22;166(3):262–77. doi: 10.1099/mic.0.000884 (PMC7376262; doi:10.1099/mic.0.000884)
Supplement: Supplementary material 1 [file mic-166-262-s001.pdf]

SUPPORTING INFORMATION

SUPPLEMENTAL BACKGROUND

Variability of pH in Contemporary Marine and Brackish Environments

At the sea surface, the annual mean pH of the ocean is pH 8.1 [1; www.noaa.gov]. However, the pH in marine environments can exhibit substantial inconstancy. Regional ocean pH values will display temporal variation due to climatic, meteorological, and seasonal perturbations [1]. The Southern Oscillation (El Niño) is a striking example. Fluctuations in the thermohaline conveyor belt, gyres, and ocean currents will also cause regional pH values to deviate [2]. For example, the pH fluxes in the Indian Ocean are quite complex, since diverse waterways (each with different chemical characteristics and hydrodynamic properties) enter this ocean from the north [1]. There is also considerable upwelling of varied water masses in the Indian Ocean. Additionally, surface water circulation is extensively reorganized after monsoons [3, 4]. In other regions, eddy currents, turbulent water flow, and front meandering can also contribute to mercurial pH, for instance the surface waters near the Strait of Magellan and Drake Passage in the Southern Hemisphere [1].

Within coastal areas, the pH variability in surface waters can be even more extensive [5]. Additionally, the pH values can also be more extreme. Submarine groundwater discharge is a hydrological process in coastal regions that can cause pH vacillations, where the seawater will sometimes be near neutral pH [5, 6]. For instance, the shoreline in Puerto Morelos, Mexico, can range from pH 7.1 to 8.0 due to an adjacent submarine spring [7]. Within kelp forests, the pH can fluctuate between pH 7.7 to 9.1 just 1 meter below the water surface in a 24-hour period [5, 8]. In estuaries, where freshwater mixes with saltwater, the hydrogen ion concentration can regularly be as low as pH 7.4. In eutrophic regions where there is great input of nutrients (salt ponds, salt marshes, and fiords), the pH can frequently be greater than pH 9.0 [5, 9]. For example, the pH

can approach pH 10.0 at Mariager Fiord in Denmark [10]. Lagoons, embayments, and tide pools with copious organic matter are other marine environments that can be especially alkaline [2, 9]. Soda lakes, which have elevated or even high salt levels, are the most alkaline aquatic environments on Earth [pH 10.0 to 12.0, 11].

Acidic measurements in marine environments are also not unusual. In some seagrass beds, observations below pH 7.0 have been recorded [5]. The rocky shore of Farland Bight in Scotland can deviate from pH 6.8 to 7.4 in the summer [2, 12]. Geological and volcanic activities near the coastline are commonly associated with acidic marine environments or at least oscillations that include values less than pH 7.0. Field sites on the coast of Ischia, Italy, typically fluctuate from pH 6.1 to 7.9 [5, 13]. The acidic pH readings observed on the Ischia coast are due to nearby underwater volcanic CO_2 vents. The ocean pH also changes with increasing depth from the water surface [14]. In the North Pacific Ocean, the pH decreases quickly through the first 1000 meters. For example, in 2006 at 25ºN, 152ºW in the North Pacific, the annual mean pH values at the surface, 500 m, and 1000 m were approximately pH 8.2, 7.5, and 7.3, respectively [14]. Salt lakes, which differ from marine lakes by being much further inland, may be as low as pH 6.0 [11]. Furthermore, marine lakes are anchialine systems [15].

SUPPLEMENTAL METHODS

Aseptic Technique and Sterile Materials

Unless otherwise explicitly stated herein, all glassware, plasticware, microfuge tubes, cryovials, scintillation vials, and containers used in this study were sterile. Pipet tips, serological pipets, microbiological media, solutions, buffers, artificial seawater, double-distilled water, and cryoprotectants were also sterile. Aseptic technique was used throughout this study. Inoculation

loops, spreaders, pestles, and other handheld microbiological instruments were either sterile or flame-sterilized with 95.0 % ethanol.

Determining the Lower and Upper pH Limits of Growth for *V. fischeri*

The pH growth limits of *V. fischeri* EM17 and EM17Tn7 were examined in FLS pH 7.0-9.0 in 0.2 pH increments. The goal was to find the lower and upper pH values that completely stopped *V. fischeri* EM17 and EM17Tn7 growth in FLS pH 7.0-9.0. To generate starter cultures, single colonies (from FLS pH 7.5 agar plates) of *V. fischeri* EM17 and EM17Tn7 were separately inoculated into test tubes containing 10.0 ml FLS pH 7.5. Liquid cultures were incubated for 12 hours at 28ºC and 200 rpm. To generate serial transfers, the *V. fischeri* starter cultures (100.0 µl) were then used to inoculate test tubes containing 9.9 ml FLS pH 7.5. Broth cultures were incubated for 3 hours at 28ºC and 200 rpm to generate log phase serial transfers. Serial transfers (100 µl) for each strain were used to inoculate replicate test tubes (n = 20) containing 9.9 ml FLS (ranging from pH 7.0-9.0 in 0.2 pH increments). The starting cell density for each strain was 5.0 X 10^5 CFUs/ml. These broth cultures were then incubated for 12 hours at 28ºC and 200 rpm. Growth for broth cultures was limited to 12 hours, because this was the incubation time for the microbial selection studies. For the microbial selection studies, the incubation period for *V. fischeri* was restricted to 12 hours before transferring into fresh media to maximize the amount of microbial evolution that occurred each day (24 hours). Since the microbial generation time slows down as a liquid culture enters late log phase and stationary phase, transferring liquid cultures into fresh media more frequently leads to more evolution in numbers of generations [16, 17].

To determine cell density (CFUs/ml), the test tube cultures were then spread onto FLS agar plates at the pH of the respective liquid culture and at pH 7.5. For example, liquid cultures

grown in FLS pH 9.0 were spread onto FLS pH 9.0 and FLS pH 7.5 agar plates. This was done to ensure pH shifts did not affect plating efficiency. Using FLS pH 7.5 agar plates for enumeration ensured that *V. fischeri* grew on solid media that was within the optimal pH range for this microorganism. Plate cultures were incubated at 28ºC for 24-48 hours. Although microbial growth and metabolism can change the pH of unbuffered media [18], *V. fischeri* growth did not change the pH of FLS broth. *V. fischeri* is a fast growing microbe with a 20-30 minute generation time in nutrient-rich media such as FLS [19]. At optimal pH, *V. fischeri* can easily reach a cell density greater than 1.0 X 10^9 CFUs/ml in FLS, FLM, and FLP within 12 hours, when the starting cell density is 5.0 X 10^5 CFUs/ml or higher.

Since *V. fischeri* EM17 and EM17Tn7 displayed growth from FLS pH 7.0-9.0, a new medium was designed with a mixture of pH buffers (MES, HEPES, EPPS, CHES, & CAPS, supplementary Table S1). This new medium was called FLM. All the buffers in FLM are "Good" biological buffers, which were characterized by N.E. Good [20, 21]. Good buffers are ideal for making solutions with buffer mixtures, when work with broad pH ranges is desirable [22, 23]. With FLM, a wider pH range (pH 5.5-11.1, supplementary Table S1) could be explored to find the low and high pH magnitudes that would completely halt *V. fischeri* EM17 and EM17Tn7 growth. The concentration of each individual buffer in FLM was 10 mM, so the total buffer concentration would not exceed 50 mM [24]. All the buffers in FLM are aminosulfonic acids, while Tris is an amine buffer [22, 25].

The pH growth limits of *V. fischeri* EM17 and EM17Tn7 were examined in FLM pH 5.4-11.0 in 0.2 pH increments. The goal was to find the lower and upper pH values that completely stopped *V. fischeri* EM17 and EM17Tn7 growth in FLM pH 5.4-11.0. To generate starter cultures, single individual colonies (from FLM pH 7.5 agar plates) of *V. fischeri* EM17 and

EM17Tn7 were separately inoculated into test tubes containing 10.0 ml FLM pH 7.5. Liquid cultures were incubated for 12 hours at 28ºC and 200 rpm. To generate serial transfers, the *V. fischeri* starter cultures (100.0 µl) were then used to inoculate test tubes containing 9.9 ml FLM pH 7.5. Broth cultures were then incubated for 3 hours at 28ºC and 200 rpm to generate log phase serial transfers.

Serial transfers (100.0 µl) for each strain were used to inoculate replicate test tubes (n = 20) containing 9.9 ml FLM (ranging from pH 5.4 to pH 11.0 in 0.2 pH increments). The starting cell density for each strain was 5.0 X 10^5 CFUs/ml. Test tube cultures were then incubated for 12 hours at 28ºC and 200 rpm. To determine cell density (CFUs/ml), the test tube cultures were then spread onto FLM agar plates at the pH of the respective liquid culture and at pH 7.5. For example, liquid cultures grown in FLM pH 6.2 were spread onto FLM pH 6.2 and FLM pH 7.5 agar plates. Plate cultures were incubated at 28ºC for 24-48 hours. *V. fischeri* growth did not change the pH of FLM. With FLM, the lower and upper pH growth limits were successfully determined for *V. fischeri* EM17 and EM17Tn7.

Microbial Growth of Derived Lines Along a pH Gradient

The acid specialists, optimists, centrists, alkaline specialists, and pH generalists were used for this experiment (Table 1). To generate starter cultures, single individual colonies (from FLM pH 7.5 agar plates) of these derived lines were separately inoculated into test tubes containing 10.0 ml FLM pH 7.5. Liquid cultures were incubated for 12 hours at 28ºC and 200 rpm. To generate serial transfers, the *V. fischeri* starter cultures (100.0 µl) were then used to inoculate test tubes containing 9.9 ml FLM pH 7.5. Broth cultures were incubated for 3 hours at 28ºC and 200 rpm to generate log phase serial transfers.

Serial transfers (100.0 µl) for each derived line were used to inoculate replicate test tubes (n = 20) containing 9.9 ml FLM pH 5.4-11.0 in 0.2 pH increments. Each of the twenty derived lines for the acid specialists, optimists, centrists, alkaline specialists, and pH generalists went into a single test tube. The starting cell density was 5.0 X 10^5 CFUs/ml. Liquid cultures were incubated for 12 hours at 28ºC and 200 rpm. To determine cell density (CFUs/ml), the test tube cultures were then spread onto FLM agar plates at the pH of the respective liquid culture and at pH 7.5. Plate cultures were incubated for 24-48 hours at 28ºC. The goal for this experiment was to compare how evolution in FLM pH 6.0, 7.4, 8.0, 10.0, and 6.0/10.0 for 2,000 generations affected the ability to grow along a pH gradient in FLM (from pH 5.4 to 11.0) relative to the ancestor.

Table S1 Seven buffers were used in the current study. The Henderson-Hasselbach Equation is used to determine the pH ranges for each buffer. Increasing the temperature to 28ºC for *V. fischeri* incubation has negligible effect on these values [22, 25]. †An aminosulfonic acid buffer. §An amine buffer. ‖An inorganic buffer.

**Buffer**	**Full Chemical Name**	**pH Range**
Tris§	Tris(hydroxymethyl)aminomethane	pH 7.0-9.0
MES†	2-(N-morpholino)ethanesulfonic acid	pH 5.5-6.7
HEPES†	4-(2-hydroxyethyl)piperazine-1-ethanesulfonic acid	pH 6.8-8.2
EPPS†	4-(2-hydroxyethyl)-1-piperazinepropanesulfonic acid	pH 7.3-8.7
CHES†	2-(cyclohexylamino)ethanesulfonic acid	pH 8.6-10.0
CAPS†	3-(cyclohexylamino)propanesulfonic acid	pH 9.7-11.1
Phosphate‖	monobasic sodium phosphate + dibasic sodium phosphate	pH 5.8-8.0

Table S2 Temperature can affect the buffer pK_a. However, the buffers used in the current study have a low degree of temperature sensitivity. The Henderson-Hasselbach Equation is used to determine pK_a with a Debye-Hückel model for electrolyte interactions [22, 26, 27]. [†]An aminosulfonic acid buffer. [§]An amine buffer. [||]An inorganic buffer.

Buffer	pK_a (4°C)	pK_a (20°C)	pK_a (25°C)	pK_a (37°C)
Tris[§]	8.7	8.2	8.1	7.7
MES[†]	6.3	6.2	6.1	6.0
HEPES[†]	7.7	7.6	7.5	7.3
EPPS[†]	8.2	8.1	8.0	7.8
CHES[†]	9.8	9.6	9.5	9.4
CAPS[†]	11.0	10.6	10.4	10.0
Phosphate[||]	7.26	7.21	7.20	7.16

SUPPLEMENTAL REFERENCES

1. **Takahashi T, Sutherland SC, Chipman DW, Goddard JG, Ho C et al.** Climatological distributions of pH, pCO_2, total CO_2, alkalinity, and $CaCO_3$ saturation in the global surface ocean, and temporal changes at selected locations. *Marine Chemistry* 2014;164:95–125.
2. **Gunderson AR, Armstrong EJ, Stillman JH**. Multiple stressors in a changing world: The need for an improved perspective on physiological responses to the dynamic marine environment. *Annual Review of Marine Science* 2016;8:357–378.
3. **Clarke A, Church J, Gould J**. Ocean processes and climate phenomena. In: G. Siedler G, Church JA, Gould J (editors). *Ocean Circulation and Climate: Observing and Modelling the Global Ocean (International Geophysics Series*. San Diego, California, United States of America: Academic Press; 2001. pp. 11-30.
4. **Gordon AL**. Interocean exchange. In: G. Siedler G, Church JA, Gould J (editors). *Ocean Circulation and Climate: Observing and Modelling the Global Ocean (International Geophysics Series*. San Diego, California, United States of America: Academic Press; 2001. pp. 303-314.
5. **Duarte CM, Hendriks IE, Moore TS, Olsen YS, Steckbauer A et al.** Is ocean acidification an open-ocean syndrome? Understanding anthropogenic impacts on seawater pH. *Estuaries and Coasts* 2013;36:221–236.
6. **Burnett WC, Bokuniewicz H, Huettel M, Moore WS, Taniguchi M**. Groundwater and pore water inputs to the coastal zone. *Biogeochemistry* 2003;66:3–33.
7. **Hofmann GE, Smith JE, Johnson KS, Send U, Levin LA et al.** High-frequency dynamics of ocean ph: a multi-ecosystem comparison. *PLOS One* 2011;6:e28983.
8. **Cornwall CE, D.; C, Hepburn CD, McGraw CM, Currie KI et al.** Diurnal fluctuations in seawater pH influence the response of a calcifying macroalga to ocean acidification. *Proceedings of the Royal Society of London B* 2013;280:20132201.
9. **Chen CY, Durbin EG**. Effects of pH on the growth and carbon uptake of marine phytoplankton. *Marine Ecology Progress Series* 1994;109:83-94.

10. **Hansen PJ**. Effect of high pH on the growth and survival of marine phytoplankton: implications for species succession. *Aquatic Microbial Ecology* 2002;28:279–288.

11. **Grant WD**. Alkaline environments and biodiversity. In: Gerday C, Glansdorff N (editors). *Extremophilies (Encyclopedia of Life Support Systems*. Oxford, United Kingdom: UNESCO/Eolss Publishers; 2006.

12. **Taylor AC**. Seasonal and diel variations of some physico-chemical parameters of boulder shore habitats. *Ophelia* 1986;25:83–95.

13. **Hall-Spencer JM, Rodolfo-Metalpa R, Martin S, Ransome E, Fine M et al.** Volcanic carbon dioxide vents show ecosystem effects of ocean acidification. *Nature* 2008;454:96–99.

14. **Byrne RH, Mecking S, Feely RA, Liu X**. Direct observations of basin-wide acidification of the north pacific ocean. *Geophysical Research Letters* 2010;6:L02601.

15. **Becking, L.E.;, Erpenbeck D, Peijnenburg KTCA, de Voogd NJ**. Phylogeography of the sponge Suberites diversicolor in Indonesia: insights into the evolution of marine lake populations. *PLOS One* 2013;8:e75996.

16. **White D, Drummond J, Clay Fuqua C**. *The Physiology and Biochemistry of Prokaryotes. 4th Edition*, Fourth ed. Oxford, United Kingdom: Oxford University Press; 2011.

17. **Gerhardt P, Drew SW**. Liquid culture. In: Gerhardt P, Murray RGE, Wood WA, Krieg NR (editors). *Methods for General and Molecular Bacteriology*. Washington, D.C., United States of America: American Society for Microbiology; 1994.

18. **Hughes BS, Cullum AJ, Bennett AF**. Evolutionary adaptation to environmental pH in experimental lineages of *Escherichia coli*. *Evolution* 2007;61:1725–1734.

19. **Soto W, Gutierrez J, Remmenga MD, Nishiguchi MK**. Salinity and temperature effects on physiological responses of *Vibrio fischeri* from diverse ecological niches. *Microbial Ecology* 2009;57:140-150.

20. **Good NE, Winget GD, Winter W, Connolly TN, Izawa S et al.** Hydrogen ion buffers for biological research. *Biochemistry* 1966;5:467–477.

21. **Good NE, Izawa S**. Hydrogen ion buffers. *Methods in Enzymology* 1972;24:53–68.

22. **Stoll VS, Blanchard JS**. Buffers: Principles and Practice. *Methods in Enzymology* 1990;182:24 – 38.

23. **Ellis KJ, Morrison JF**. Buffers of constant ionic strength for studying pH-dependent processes. *Methods in Enzymology* 1982;87:405-426.

24. **Ferguson WJ, Braunschweiger KI, Braunschweiger WR, Smith JR, McCormick JJ et al.** Hydrogen Ion Buffers for Biological Research. *Analytical Biochemistry* 1980;104:300–310.

25. **Ferreira CMH, Pinto ISS, Soares EV**. (Un)suitability of the use of pH buffers in biological, biochemical and environmental studies and their interaction with metal ions – a review. *Royal Society of Chemistry Advances* 2015;5:30989–31003.

26. **Debye P, Hückel E**. The theory of electrolytes: I. lowering of freezing point and related phenomena. *Physikalische Zeitschrift* 1923;24:185–206.

27. **Blanchard JS**. Buffers for enzymes. *Methods In Enzymology* 1984;104:404-414.
